# Alterations in mucosa branched *N*-glycans lead to dysbiosis and downregulation of ILC3: a key driver of intestinal inflammation

**DOI:** 10.1080/19490976.2025.2461210

**Published:** 2025-02-07

**Authors:** Cláudia S. Rodrigues, Joana Gaifem, Márcia S. Pereira, Maria Francisca Alves, Mariana Silva, Nuno Padrão, Bruno Cavadas, Catarina Moreira-Barbosa, Inês Alves, Ricardo Marcos-Pinto, Joana Torres, Aonghus Lavelle, Jean-Frederic Colombel, Harry Sokol, Salomé S. Pinho

**Affiliations:** a Institute for Research and Innovation in Health (i3S), Immunology, Cancer & Glycomedicine Group, University of Porto, Porto, Portugal; b School of Medicine and Biomedical Sciences (ICBAS), University of Porto, Porto, Portugal; c Faculty of Sciences, University of Porto, Porto, Portugal; d Faculty of Medicine, University of Porto, Porto, Portugal; e Hospital da Luz Learning Health, Luz Saúde, Lisbon, Portugal; f Department of Gastroenterology, Centro Hospitalar do Porto, Porto, Portugal; g Centro de Investigação em Tecnologias e Serviços de Saúde, University of Porto, Porto, Portugal; h Division of Gastroenterology, Hospital Beatriz Ângelo, Loures, Portugal; i Faculty of Medicine, University of Lisbon, Lisbon, Portugal; j Division of Gastroenterology, Hospital da Luz, Lisbon, Portugal; k Centre de Recherche Saint-Antoine, CRSA, AP-HP, Saint-Antoine Hospital, Gastroenterology Department, Sorbonne Université, INSERM, Paris, France; l Henry D. Janowitz Division of Gastroenterology, Department of Medicine, Icahn School of Medicine at Mount Sinai, New York, NY, USA; m INRAE, AgroParisTech, Micalis Institute, Université Paris-Saclay, Jouy-en-Josas, France; n Paris Center for Microbiome Medicine (PaCeMM) FHU, Paris, France

**Keywords:** *N*-glycans, intestinal inflammation, microbiome, ILC3, prophylaxis

## Abstract

The perturbation of the symbiotic relationship between microbes and intestinal immune system contributes to gut inflammation and Inflammatory Bowel Disease (IBD) development. The host mucosa glycans (glycocalyx) creates a major biological interface between gut microorganisms and host immunity that remains ill-defined. Glycans are essential players in IBD immunopathogenesis, even years before disease onset. However, how changes in mucosa glycosylation shape microbiome and how this impact gut immune response and inflammation remains to be clarified. Here, we revealed that alterations in the expression of complex branched *N*-glycans at gut mucosa surface, modeled in glycoengineered mice, resulted in dysbiosis, with a deficiency in Firmicutes bacteria. Concomitantly, this mucosa *N*-glycan switch was associated with a downregulation of type 3 innate lymphoid cells (ILC3)-mediated immune response, leading to the transition of ILC3 toward an ILC1 proinflammatory phenotype and increased TNFα production. In addition, we demonstrated that the mucosa glycosylation remodeling through prophylactic supplementation with glycans at steady state was able to restore microbial-derived short-chain fatty acids and microbial sensing (by *NOD2* expression) alongside the rescue of the expression of ILC3 module, suppressing intestinal inflammation and controlling disease onset. In a complementary approach, we further showed that IBD patients, often displaying dysbiosis, exhibited a tendency of decreased *MGAT5* expression at epithelial cells that was accompanied by reduced ILC3 expression in gut mucosa. Altogether, these results unlock the effects of alterations in mucosa glycome composition in the regulation of the bidirectional crosstalk between microbiota and gut immune response, revealing host branched *N*-glycans/microbiota/ILC3 axis as an essential pathway in gut homeostasis and in preventing health to intestinal inflammation transition.

## Introduction

The human gut is home to a diverse collection of microorganisms – the intestinal microbiota – that has co-evolved with the immune system, maintaining a symbiotic relationship essential for homeostasis.^1^ The perturbation of the cooperative relationship between microbes and the intestinal immune system is shown to contribute to the onset of many inflammatory diseases, such as Inflammatory Bowel Disease (IBD),^2,3^ an event that can occur years before diagnosis.^4^ Despite advances in therapeutic resources, IBD remains incurable.^5^ Therapies modulating microbiota are an emerging field of research,^6,7^ with potential to revolutionize IBD therapy in a near future. Thus, it is essential to understand the causes underlying the metabolic changes associated with loss of gut microbial equilibrium (dysbiosis) and with gut inflammation, envisioning novel disease biomarkers and new therapeutic targets.

Host glycocalyx, which is the repertoire of glycans/sugar chains expressed at the surface of all cells, is a major biological and physical interface between intestinal mucosa, microorganisms and host immune response, essential to guarantee homeostasis.^8–10^ Hence, host glycocalyx is a key target in a disease context that remains largely unexplored. In fact, the dense and complex coat of sugar-chains that cover our gut mucosa is an essential niche for microbiota colonization.^11^ Moreover, the human gut microbiota is able to degrade and use host glycans as a major source of nutrients and energy,^12,13^ producing short-chain fatty acids (SCFAs) that are fundamental in maintaining intestinal homeostasis through the regulation of epithelial and immune cells.^2,14,15^

Mucins are the typical example of mucosa glycoproteins, heavily decorated with *O*-glycans, which are essential for building barriers that protect our inner surfaces from bacteria.^16,17^ Changes in the expression of truncated mucins *O*-glycans or the disruption of epithelial fucosylation were associated with increased susceptibility to bacterial infection.^18^ However, the diversity of glycans presentation at the gut mucosa is enormous, going far beyond mucin *O*-glycans. The prominent expression of *N*-glycan structures in the human intestine places them as ideal, hitherto poorly defined, molecular interfaces between microorganisms and the immune microenvironment.^19^ However, whether and how *N*-glycans at the surface of gut mucosa act as shapers of the gut microbiota composition and function and how this impact in the breakdown of gut homeostasis associated with IBD immunopathogenesis is a fundamental question that remains unanswered.

Previous evidence from our group have been demonstrating the key role of protein glycosylation in IBD immunopathogenesis, revealing the power of glycome alterations even years before disease diagnosis.^8,20–23^ We showed that ulcerative colitis patients exhibit a deficiency in the expression of complex branched *N*-glycans structures, associated with T cell hyperactivity and disease severity.^23^ We also demonstrated that mice deficient in *Mgat5* glycogene, thus lacking *N*-acetylglucosaminyltransferase-V (GnT-V)-mediated complex branched *N*-glycans structures, have an increased susceptibility to severe forms of colitis and to early onset disease.^20^ Importantly, changes in glycome were found to occur years before Crohn´s disease (CD) diagnosis, supporting the predominant biological relevance of glycans in health to intestinal inflammation transition and IBD onset.^21^ In addition, alterations in mucosa glycosylation have been also correlated with risk for colitis-associated cancer development.^22,24–26^ Altogether, this previous evidence highlights the prominent role of *N*-glycans in modulating gut immunity, with promising translational clinical applications in IBD prognosis and treatment.^22,27,28^ However, when, why and how an altered host mucosa glycome shapes microbiome composition associated with immune activation and IBD development is a key question that remains ill-defined. In this study, we created a glycoengineered mice deficient in *Mgat5* gene to investigate whether and how an altered host glycocalyx, translated into fluctuations in the abundance and spatial distribution of mucosa *N*-glycans, is a fundamental factor that mediates loss of immune-tolerance, by leading to the selective overgrowth of pathobionts, triggering dysbiosis and consequently the activation of intestinal immune response. Our results reveal host glycocalyx and specifically mucosa branched *N*-glycans as master regulators at the frontier of microbiome and ILC3/ILC1-mediated immune response, bringing to light the power of glycosylation reprogramming of gut mucosa as a promising intervention strategy to prevent IBD onset with clinical applications.

## Materials and methods

### Human cohort

To characterize the immune populations, fresh intestinal biopsies from 5 patients diagnosed with IBD (*n* = 2 UC; *n* = 3 CD) and 3 healthy controls were retrieved at Centro Hospitalar Universitário Santo António, Porto, Hospital Beatriz Ângelo, Loures, and Hospital da Luz, Lisbon. Healthy individuals are those without significant pathological findings. Samples were taken from the terminal ileum or the sigmoidal colon. In IBD patients, both inflamed and non-inflamed material were collected (See Supplementary Table S1). Patient identity was taken properly into account to allow for dependent samples in the statistical analyses. Ethical approval was obtained at the Ethical Committees of all Hospitals. All study participants gave written informed consent prior to sampling and data collection.

### Animals

C57BL/6 wild-type (*Mgat5*^WT^) and *Mgat5*-deficient mice (*Mgat5*^−/−^, kindly provided by Michael Pierce, University of Georgia, Athens, GA) were bred and maintained in accredited animal facilities at the Institute for Research and Innovation in Health (i3S). Mice were housed in groups of 5–10, in HEPA filter-bearing cages, under 12-hour light/dark cycles. Autoclaved chow and water were provided *ad libitum*. Cages were enriched with nesting materials. Females between 9 and 14 weeks were used. Experiments were conducted with the approval of the Animal Ethics Committee and the Animal Welfare Body of the i3S. The protocols are in agreement with in-house standards and rules for animal experimentation, comply with national legislation and are integrated within a project, which is licensed by the Portuguese competent authority, DGAV (license number 009268/2022-06-02).

### Co-housing

Four-week-old *Mgat5*^WT^ and *Mgat5*^−/−^ mice were weaned and moved to the same cage, in order to share the same environment. Mice were housed in the same cage at a maximum amount of 10 mice per cage. Co-housing was performed for 5 weeks. Stool samples were collected before and after co-housing for both *Mgat5*^WT^ and *Mgat5*^−/−^ mice.

### GlcNAc supplementation

To assess the prophylactic properties of glycan supplementation, 4-week-old *Mgat5*^WT^ and *Mgat5*^−/−^ mice were treated with 800 mg/Kg/day of GlcNAc (Wellesley Therapeutics, Inc.) in the drinking water, *ad libitum*, for 8 weeks. This dose was based on previous publications from others and us.^20,29–31^ No alterations in the regular behavior and water consumption were observed. Stool samples were collected before and after GlcNAc supplementation. The histological profile, as well as the immune response, were analyzed upon GlcNAc treatment.

### DSS-induced colitis

Mice with 9 to 11 weeks of age were given dextran sulfate sodium (DSS; 2% (w/v), molecular weight approximately 36,000–50000 Da; MP Biomedicals) in the drinking water *ad libitum* for 7 days. Clinical signs of colitis, such as weight loss, stool consistency, and presence of blood were monitored daily and measured by the disease activity index (DAI) (See Supplementary Table S2). Mice were euthanized at the end of each experiment or earlier, if the symptoms of clinical disease reached one of these endpoints: more than 20% weight loss, diarrhea, or gross bleeding. The histological profile, as well as the immune response, were analyzed after colitis induction.

### Isolation of lamina propria leukocytes (LPL)

To isolate lamina propria leukocytes (LPL), colons were flushed with Ca- and Mg-free PBS Fragments of 0.5–1 cm were incubated in RPMI 1640 medium supplemented with 10% fetal bovine serum (FBS), 1% penicillin/streptomycin, 1 mm CaCl_2_, 1 mm MgCl_2_, and 1 mg/mL of collagenase IV (Sigma), under 100 rpm agitation at 37°C for 40 minutes. Tissues were dissociated and filtered through a 70 µm cell strainer (BD Biosciences). Cell suspension was centrifuged, the pellet was resuspended in RPMI 1640 medium supplemented with 10% FBS, and 1% penicillin/streptomycin, and layered upon Lymphoprep solution in a proportion of 1:2 (Lymphoprep:cell suspension). After gradient centrifugation at 800 g for 20 minutes at 20°C (without acceleration or break), cells retained in the interface were collected for the staining of immune cells, whereas cells retained in the bottom of the tube were collected for staining of nonimmune cells. Both fractions were washed in RPMI and kept in ice before analysis.

To isolate lamina propria leukocytes from human fresh colonic biopsies it was performed a mechanical dissociation in HBSS with 1% Pen/Strep and 0.1% Gentamycin. Then, human fresh colonic biopsies were digested with 0.9 mg/mL of collagenase IV in RPMI supplemented with 10% FBS, 100 U/mL Pen/Strep, 1 mm CaCl_2_ and 1 mm MgCl_2_, for 45 min with agitation, at 37°C. Cell suspensions were then filtered in 70 μm cell strainers and washed with PBS. Following, cells were centrifuged for 10 min at 300 g and 4°C, and the pellet was resuspended in FACS buffer and ready to proceed for flow cytometry staining.

### Flow cytometry analysis

All cells analyzed by flow cytometry were stained with Fixable Viability Dye (FVD) eFluor™ 780 for viability analysis and exclusion of dead cells. For lectin staining, cells were incubated for 15 minutes, at 4°C protected from light, with conjugated lectins: L-PHA-fluorescein, L-PHA-biotin, GNA-fluorescein, Sambucus Nigra Lectin (SNA)-fluorescein, Maackia Amurensis Lectin II (MAL-II)-biotin and Ulex Europaeus Agglutinin I (UEA-I)-biotin, for evaluation of complex branched *N*-glycans (L-PHA), high-mannose residues (GNA), α-2,6 sialic acid (SNA), α-2,3 sialic acid (MAL-II), and α-linked fucose residues (UEA-I). Cells stained with biotinylated lectins were then incubated with streptavidin-PE or streptavidin-Spark Blue 550 for 30 minutes.

For staining of lamina propria lymphocytes, cells were stimulated with 20 ng/mL of phorbol myristate acetate (PMA), 200 ng/mL ionomycin calcium salt, and 10 µg/mL of brefeldin A for 4 hours at 37°C. After staining with FVD, surface staining was performed by incubation for 30 minutes at 4°C with the surface antibodies shown in Supplementary Table S3. Intracellular staining was performed using the eBioscience Foxp3/Transcription Factor Staining Buffer set and the intracellular antibodies displayed in Supplementary Table S3. Gating strategy is presented in Supplementary Figure S2A. Cells analysis was performed on a BD FACSCanto II (Becton Dickinson) or Cytek Aurora (Cytek). Data was analyzed using FlowJo software (Tree Star).

### Tissue histochemistry and histological characterization

Samples from colons were fixed in 4% paraformaldehyde and 5 µm paraffin-embedded sections were stained with hematoxylin and eosin (H&E). Inflammation was assessed blindly using a graduated semi quantitative system as previously described.^32^ Besides H&E staining, lectin histochemistry was performed to assess the glycan profile of the colonic tissue. Paraffin-embedded sections were incubated with lectins *Phaseolus vulgaris* Leucoagglutinin (L-PHA) and *Galanthus nivalis* Lectin (GNA) for evaluation of complex branched *N*-glycans (L-PHA) and high-mannose residues (GNA). Staining with Alcian Blue/Periodic Acid-Schiff (AB/PAS) of colon section was performed to evaluate polysaccharide structures and mucus layer. Goblet cell number was assessed for each experimental condition in a blinded fashion. Only intact crypts, cut longitudinally from crypt opening to bottom, were quantified. The mucus depth for each section was measured on 3 different areas of microscopic images captured by a digital camera, under a microscope using a 100× objective lens. Images were captured using an Brightfield Microscope – Leica DM2000 LED microscope and recorded with a digital camera Flexacam C3 using Leica Application Suite X (LAS X) software. Image analysis was performed using Fiji (ImageJ) software.

### Intestinal permeability assay

*In vivo* intestinal permeability was assessed by administration of fluorescein isothiocyanate (FITC) labeled dextran. Food and water were withdrawn for 8 hours. Mice were administered 44 mg/100 g of body weight of FITC-labeled dextran (TdB Labs AB; 4 kDa) by oral gavage. Serum was collected four hours later, and fluorescence intensity was measured by spectrophotofluorimetry (excitation: 485 nm; emission: 528 nm).

### RNA extraction, cDNA synthesis and quantitative real-time PCR

Total RNA from mice was extracted using RNAqueous-Micro Kit (Invitrogen) according to the manufacturer’s protocol. Total RNA was quantified using the Nanodrop 1000 system and RNA was transcribed into single-stranded cDNA using Superscript IV Reverse Transcriptase (Invitrogen) and Random Hexamer Primers (Invitrogen, Oregon, USA) according to the manufacturer’s recommendations. The quantitative real-time PCR (qRT-PCR) was performed in 96-well reaction plates. cDNA was amplified using NZYSupreme qPCR Green Master Mix, ROX (NZYtech) using specific using the primers stated on Supplementary Table S4. Amplification data were acquired with 7500 Fast Real-Time PCR System (Applied Biosystems). Glyceraldehyde 3-phosphate dehydrogenase (*Gapdh*) were used as housekeeping gene. Relative quantification values for gene expression were calculated based on the ΔCt method, as follows: 2^−(Target gene mRNA expression – housekeeping gene mRNA expression)^.

### Cytokine quantification

For the colon explants, cytokine concentrations were analyzed by flow cytometry using cytometric bead arrays: the LEGENDplex™ MU Th Cytokine Panel (13-plex) (Biolegend), according to the manufacturer’s instructions. Samples were measured on the BD Accuri C6 instrument (BD Biosciences, US) using a specific template provided by BD Biosciences.

### SCFAs quantification

Colonic tissue was minced to small pieces and homogenized in 500 µL of cold PBS with a glass homogenizer on ice. The suspension was ultrasonicated followed by a centrifugation for 15 minutes at 5000 rpm. The quantitative determination of SCFAs in colonic tissue was determined using the Mouse Short-Chain Fatty Acids (SCFAs) ELISA kit (Amsbio), according to the manufacturer’s instructions. Optical Density (O.D.) was measured at 450 nm using a microplate reader (Biotek Instruments).

### Bioinformatic analysis

#### 16S rRNA sequencing, bioinformatic and statistical analysis of gut microbiota composition

Stools were collected before DSS treatment from mice, single-housed and co-housed, as well as before and after GlcNAc supplementation, and snap frozen in liquid nitrogen. Microbiota composition was determined by 16S rRNA gene sequencing and analyzed as previously described.^33^ Sequencing was performed on an Illumina MiSeq platform (Illumina) at GenoScreen with a 250-bp paired-end sequencing protocol. Raw paired-end reads were subjected to the following process: (1) quality filtering using the PRINSEQ-lite PERL script^34^ by truncating the bases from the 3′ end that did not exhibit a quality & less than 30 based on the Phred algorithm; (2) paired-end read assembly using FLASH (fast length adjustment of short reads to improve genome assemblies) with a minimum overlap of 30 bases and a 97% overlap identity; and (3) searching and removing both forward and reverse primer sequences using CutAdapt, with no mismatches allowed in the primers sequences. Assembled sequences without forward and reverse primers were removed.

Joined sequences were imported into the amplicon version of Qiime2 (version 2023.9).^35^ Sequence inference was performed using Deblur.^36^ Taxonomy was assigned using the SILVA database (v138.1).^37^ Functional inference was performed using Picrust2 (v2.4.1).^38^ All downstream analysis was performed in R and RStudio. For samples from the animal experiment with glycan supplementation (Supplementary Figure S5F), forward reads only were analyzed using the dada2 package.^39^ Taxonomic processing and downstream analysis were otherwise the same. The phyloseq package (v1.40.0) was used to handle and analyze microbiome data.^40^ The vegan package (v2.6.2) was used to perform diversity analyses. Samples were rarefied to an even depth of the sample with the lowest counts prior to performing diversity analyses. Alpha diversity was calculated using the Chao1 and Shannon indices and beta diversity was calculated using the unweighted unifrac distance.^41^ Pairwise differential abundance testing was performed between single-housed wild type vs single-housed *Mgat5*^−/−^, as well as *Mgat5*^−/−^ single-housed vs co-housed animals with taxa agglomerated at the genus level and rarefied, as recently suggested,^42^ using MaAsLin 2,^43^ with the variable of interest a fixed-effect and the experiment as a random effect. “TSS” normalization and “LOG” transformation was used with prevalence filtering at 0.1. A q-value of 0.2 was applied to filter taxa. A similar analysis was performed using the unstratified pathway output of Picrust2 (*Mgat5*^−/−^ single-housed vs co-housed animals), without prior rarefaction and using a q-value of 0.05 and prevalence filtering of pathways in at least 50% of samples. For correlation of disease activity and differentially abundant taxa between *Mgat5*^−/−^ single-housed vs co-housed animals, the area-under-the-curve (calculated using the AUC function in the DescTools package (v0.99.54) with the “trapezoid” method) was calculated between day 0 and day 10 (with day 7 removed as some measurements were missing). Taxa were proportion-normalized, and the spearman correlation coefficient was used. Samples with a correlation p-value of <0.05 were manually plotted with the ‘stat_smooth’ function in ggplot2 using a “gam” method. All plotting was performed using ggplot2 (v3.4.1) and ggpubr (v0.4.0). Statistical tests of pairwise comparisons between independent groups were performed using the Wilcoxon rank sum test.

### Human single-cell and bulk RNA sequencing analysis

Single cell RNA-seq data from Martin et al.^44^ with the accession number GSE134809 was downloaded from NCBIs GEO database. Cell type annotations provided by the authors were used for subsequent analysis. For the quantification of *MGAT5* in the epithelial component, the mean of all cells with above zero expression, per patient, was calculated. To analyze the epithelial component, we evaluated all passing cells excluded from the study.^44^ Passing cells were considered all those with more than 800 UMIs, a percentage of mitochondrial genes below 25% and a percentage of hemoglobin genes lower than 10%. All analysis were performed using Seurat v5.1.0. To focus only on the epithelial component, mesenchyme, immune, and hematopoietic cells were removed based on marker genes *LSP1*, *MZB1*, *VIM*, *CD52*, and *COL3A1*. Only patients with more than 50 cells were kept. After the quality control step, library size normalization was performed using Seurat Normalize Data. Highly variable genes were identified by fitting the mean-variance relationship and dimensionality reduction was performed using principal-component analysis. Scree plots were used to determine principal components to use for clustering analyses. Cells from different patients were merged and batch effects were corrected using harmony (version 1.2.0) algorithm. Cells were then clustered using the Leiden algorithm for modularity optimization using kNN graph as input, for a cluster resolution of 0.8. Cell clusters were visualized using UMAP algorithm with the first 20 dimensions as input. Both clustering and UMAP algorithms used the harmony dimensionality reduction as input instead of principal components. Cell-type annotation was performed based on the expression profile of marker genes from published work on human intestinal tract.^45^ Briefly, all EPCAM-positive cells were divided into stem cells (*LGR5*, *ASCL2*, *SMOC2*, *RGMB*, *OLFM4*), Paneth (*DEFA5*, *DEFA6*, *REG3A*), cycling transit-amplifying (*MKI67*, *TOP2A*, *PCNA*), goblet cells (*CLCA1*, *SPDEF*, *FCGBP*, *ZG16*, *MUC2*), BEST4 enterocytes (*BEST4*, *OTOP2*, *CA7*), enterocytes (*RBP2*, *ANPEP*, *FABP2*) and colonocytes (*CA2*, *SLC26A2*, *FABP1*), enteroendocrine cells (*EEC*; *CHGA*, *CHGB*, *NEUROD1*), Tuft cells (*POU2F3*, *LRMP*, *TRPM5*). Gene signatures were evaluated using UCell (version 2.6.2) for a set GlcNAc transferases participating in the branching *N*-glycosylation pathway (*MGAT5*, *MGAT5B MGAT4D*, *MGAT4C*, *MGAT4B*, *MGAT4A*, *MGAT3*, *MGAT1*). Significance was calculated using the Wilcoxon test. Percentage of ILC1 and ILC3 was calculated based on the total number of cells within each patient.

Bulk RNA sequencing from 12 healthy controls and 9 Crohn disease with inflamed epithelial were retrieved from dataset published by Häsler et al.^46^ Violin plots using Deseq2 normalized counts of *MGAT5* and *RORC* were generated to assess differences in expressions amongst groups.

### Statistical analysis

All statistical analyses were executed using GraphPad Prism 8.0 Software. Data from flow cytometry was analyzed with Flow Cytometry Analysis Software (FlowJo). The presence of outliers was evaluated using ROUT method (Q = 1%). Normality was assessed by performing D’Agostino-Pearson or Shapiro-Wilk normality tests. For statistical analyses, unpaired *t*-test or Mann-Whitney test were used, according to normality. For DAI analysis, two-way ANOVA was used. Data obtained were expressed as mean ± standard deviation (SD). Statistical significance was considered at *p* ≤ 0.05 and statistically significant values were represented as follows: *p ≤ 0.05, **p ≤ 0.01, ***p ≤ 0.001 and ****p ≤ 0.0001.

## Results

### *Changes in mucosa branched* N-*glycosylation promote gut dysbiosis and increase intestinal permeability associated with intestinal inflammation*

**Figure 1.**
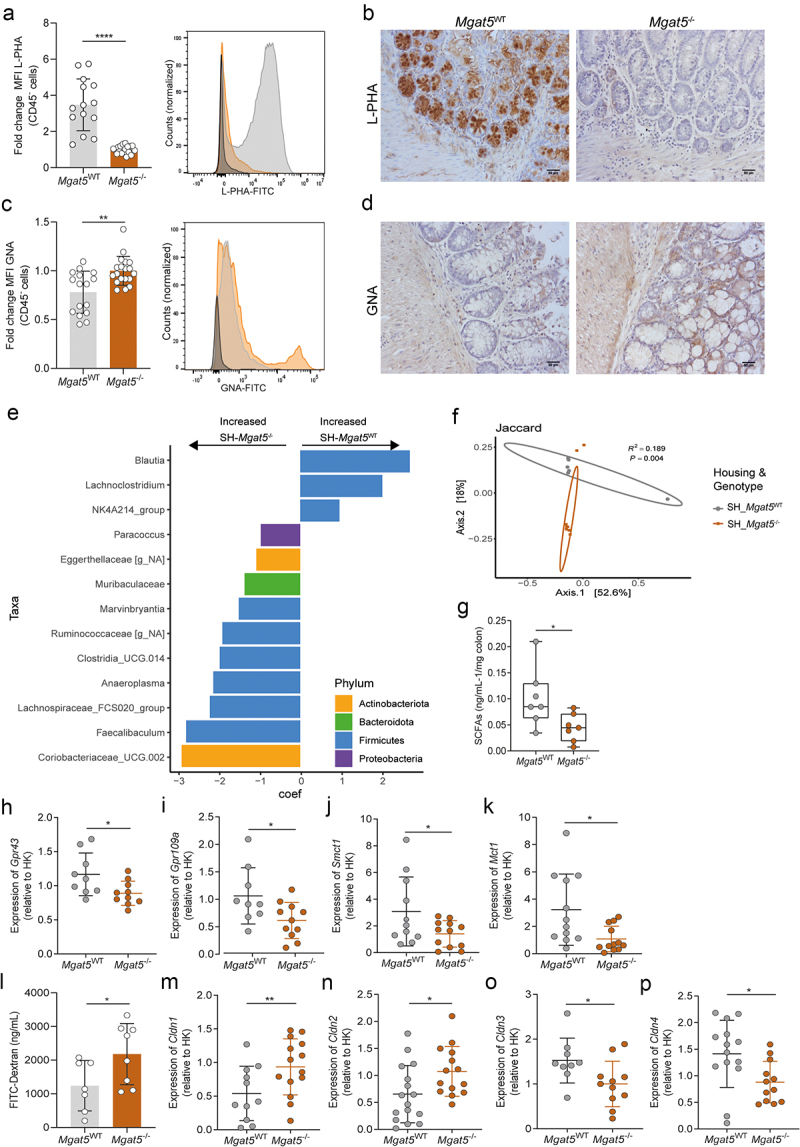
Reduction of branched *N*-glycans in mice promotes intestinal permeability and gut dysbiosis. (a and b) levels of β1,6-branching *N*-glycans at steady state in epithelial cells (CD45- cells) from *Mgat5*^−/−^ mice and *Mgat5*^WT^ controls. (a) L-PHA lectin was used to detect branched *N*-glycans and the median fluorescence intensity (MFI) was determined by flow cytometry. MFI was normalized for the average of *Mgat5*^−/−^ mice MFI; in the representative histogram, dark gray, light gray and orange depicts unstained control, *Mgat5*^*WT*^ and *Mgat5*^*-/-*^, respectively. (b) Lectin histochemistry staining with L-PHA in mouse colonic samples. Scale bar = 50 μm. (c and d) levels of high-mannose *N*-glycans at steady state in epithelial cells (CD45^−^ cells) from *Mgat5*^*-/-*^ mice and *Mgat5*^WT^ controls. (c) GNA lectin was used to detect mannose *N*-glycans and the median fluorescence intensity (MFI) was determined by flow cytometry. MFI was normalized for the average of *Mgat5*^−/−^ mice MFI; in the representative histogram, dark gray, light gray and orange depicts unstained control, *Mgat5*^*WT*^ and *Mgat5*^*-/-*^, respectively. (d) Lectin histochemistry staining with GNA in mouse colonic samples. Scale bar = 50 μm. (e) Linear discriminant analysis (LDA) of the gut microbiota composition based on 16S rRNA sequencing in the fecal samples from *Mgat5*^*-/-*^ and *Mgat5*^WT^ mice at steady state. (f) Principal component analysis (PCoA) of gut microbiota composition generated on Jaccard based on 16S rRNA sequencing of fecal samples from *Mgat5*^*-/-*^ and *Mgat5*^WT^ mice at steady state. (g) Concentration of short-chain fatty acids (SCFAs) measured by ELISA in the colon of *Mgat5*^*-/-*^ and *Mgat5*^WT^ mice at steady state. (h-k) the mRNA expression levels at steady state of genes encoding SFCAs receptors (h) Gpr43, (i) Gpr109a, and SFCAs transporters (j) Smct1 and (k) Mct1 in the colonic tissue from *Mgat5*^*-/-*^ and *Mgat5*^WT^ mice measured by RT-qPCR. Expression of target gene mRNA was calculated based on housekeeping gene (*Gapdh*). mRNA expression levels were normalized for the average of mRNA levels of *Mgat5*^−/−^ mice. (l) Intestinal permeability measured by FITC-labeled dextran in Mgat5^−/−^ and *Mgat5*^WT^ mice at steady state. (m-p) the mRNA expression levels at steady state of genes encoding for (m) claudin-1, (n) claudin-2, (o) claudin-3, and (p) claudin-4 in the colonic tissue from *Mgat5*^*-/-*^ mice and *Mgat5*^WT^ controls measured by RT-qPCR. Expression of target gene mRNA was calculated based on housekeeping gene (*Gapdh*). mRNA expression levels were normalized for the average of mRNA levels of *Mgat5*^−/−^ mice. (a and c) *n* = 14–19 per group. (f) *n* = 6–7 per group. (g) *n* = 7 per group. (h-k) *n* = 9–12 per group. (l) *n* = 7–8 per group (m-p) *n* = 9–16 per group. Each datapoint represents an individual animal. Data is represented as mean ± SD. *p < 0.05; **p < 0.01; ****p < 0.0001 using an unpaired two-tailed Student’s *t*-test or Mann-Whitney test.

**Figure 2.**
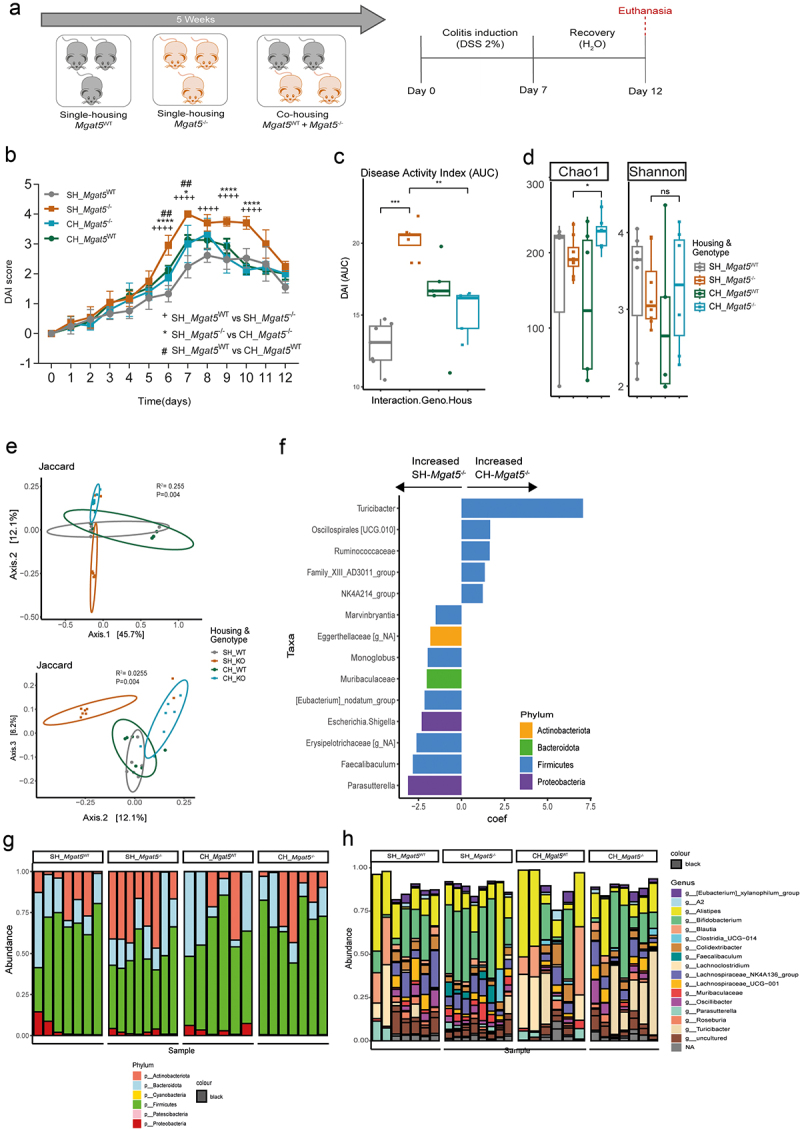
Co-housing of *Mgat5*^−/−^ mice with *Mgat5*^WT^ mice impacts gut dysbiosis and colitis susceptibility. (a) Schematic representation of co-housing experiment. *Mgat5*^*-/-*^ and *Mgat5*^WT^ mice (controls) were co-housed for 5 weeks. Then, colitis was induced by giving *ad libitum* 2% DSS, in the drinking-water, for 7 days followed by normal water for 5 additional days. (b) Disease activity score (DAI) of single-housed (SH) and co-housed (CH) *Mgat5*^*-/-*^ or *Mgat5*^WT^ mice upon DSS-induced colitis. (c) The area-under-the-curve (AUC) of DAI between single-housed (SH) and co-housed (CH) *Mgat5*^*-/-*^ or *Mgat5*^WT^ mice upon DSS-induced colitis. (d) Richness and evenness of fecal microbiota composition from mice before and after co-housing analyzed by 16S rRNA sequencing. (e) Principal component analysis (PCoA) of gut microbiota composition generated on Jaccard based on 16S rRNA sequencing. (f) Linear discriminant analysis (LDA) of the gut microbiota composition based on 16S rRNA sequencing in the fecal samples of single-housed (SH) and co-housed (CH) *Mgat5*^*-/-*^ at steady state. (g and h) phyla and genus level gut microbiota composition analysis based on 16S rRNA sequencing in the fecal samples of single-housed (SH) and co-housed (CH) *Mgat5*^*-/-*^ or *Mgat5*^WT^ mice. (B-H) *n* = 5–8 per group. Each datapoint represents an individual animal. Data is represented as mean ± SD. *p < 0.05; **p < 0.01; ****p < 0.0001 using two-way ANOVA or Wilcoxon rank sum test.

Our previous evidence showed that the deficiency in the expression of complex branched *N*-glycans in *Mgat5* knockout mice (*Mgat5*^*-/-*^) resulted in an early onset colitis in DSS-induced model, with mice developing severe forms of intestinal inflammation (Supplementary Figure S1A).^20^ However, whether this deficiency on mucosa glycome may impact microbiota composition associated with colitis susceptibility remains unknown. An extensive characterization of the mucosa glycosylation profile was performed in *Mgat5*^*-/-*^ mice using lectins that specifically recognize different glycan structures (Supplementary Figure S1B). The results showed the clear deficiency in β1,6-GlcNAc complex branched *N*-glycans (detected by L-PHA) in the intestinal epithelial cell surface (CD45^−^ cells) of colitis-susceptible *Mgat5*^*-/-*^ mice compared with *Mgat5*^WT^ (Figure 1(a,b); Supplementary Figure S1C and D). In contrast, higher levels of less complex, mannose-enriched *N*-glycans (GNA binding) at the gut mucosa surface were detected in *Mgat5*^*-/-*^ mice (Figure 1(c,d); Supplementary Figure S1C and E) in comparison with *Mgat5*^WT^. These observations are supported by the lower ratio of LPHA/GNA exhibited by *Mgat5*^*-/-*^ mice (Supplementary Figure S1F), which points toward an overexposure of mannose-enriched epitopes at the gut mucosa surface. A slight decrease in α(1,2)-fucosylation (UEA-I binding) (Supplementary Figure S1G) was observed and no alterations in terminal α2,6 sialylation (SNA binding) (Supplementary Figure S1H) or α(2,3) linked sialic acid (MAL II binding) (Supplementary Figure S1I) in epithelia were detected. Despite the fact that mucus layer is mainly *O*-glycosylated, the presence of *N*-glycans may also affect mucins folding and dimerization.^47^ Thus, the impact of the deficiency in β1,6-GlcNAc complex branched *N*-glycans in mucus layer of *Mgat5*^*-/-*^ mice was analyzed, showing that the number of goblet cells seems to be reduced in *Mgat5*^*-/-*^ mice relative to *Mgat5*^WT^ (Supplementary Figure S1J). Moreover, it was also observed that the colonic mucus layer of *Mgat5*^*-/-*^ mice appears to be much thinner than *Mgat5*^WT^ (Supplementary Figure S1K). Then, we evaluated how the mucosa glycan switch imposed by the deficiency complex branched *N*-glycans and overexposure of mannose-enriched glycans affects the gut microbiota composition, by performing 16S rRNA sequencing of stool content from *Mgat5*^−/−^ mice and *Mgat5*^WT^ mice in steady state. The results showed that mice lacking complex branched *N-*glycans display a clear dysbiotic microbiota composition at steady state when compared with *Mgat5*^WT^ mice (Figures 1(e,f) and 2e). Indeed, *Mgat5*^−/−^ mice showed an imbalance in the composition of different phyla relative to *Mgat5*^WT^ mice with significantly decreased abundance of specific taxa belonging to Firmicutes phylum, such as *Blautia, NK4A214 group* and *Lachnoclostridium* genera. In contrast, the increased abundance of bacterial species such as *Marvinbryantia, Ruminococcaseae, Clostridia_UCG 014, Anaeroplasma, and Lachnospiraceae_FCS020_group*, were detected in *Mgat5*^*-/-*^ mice. Additionally, *Muribaculaceae* family from Bacteroidota phylum, *Coriobacteriaceae*_*UCG 002*, *Eggerthellaceae* from Actinobacteriota phylum and *Paracoccus* genus belonging to Proteobacteria phylum, also exhibited increased abundance in *Mgat5*^*-/-*^ mice (Figure 1(e)). Accordingly, we also found that the colonic microenvironment of *Mgat5*^−/−^ mice exhibited significant decreased levels of SCFAs, that are considered major players in intestinal homeostasis^48^ (Figure 1(g)). In line with this, we also demonstrated a concomitant reduced expression of receptors and transporters of SCFAs,^49^ namely *Gpr43*, *Gpr109a*, *Mct1* and *Smct1* (Figure 1(h–k)).

Given the biological relevance of intestinal glycocalyx in maintaining the integrity of gut barrier, we then evaluated the impact of the deficiency of complex branched *N*-glycans in intestinal permeability. By performing FITC-dextran assay, we showed that *Mgat5*^*-/-*^ mice exhibited an increased permeability of intestinal epithelial barrier, as illustrated by the increased FITC-dextran levels detected in *Mgat5*^−/−^ mice serum (Figure 1(l)). In addition, and since the regulation of tissue integrity and gut permeability also depends on the expression and interaction of proteins in cell – cell junctional complexes, such as the tight-junctions,^50^ we also evaluated the expression of claudin-1, −2, −3, −4 and occludin. The results showed an increased expression of claudin-1 and −2 in *Mgat5*^−/−^ mice (Figure 1(m,n)), as described to occur in IBD,^51,52^ alongside a marked loss of sealing claudins −3 and −4 in comparison with *Mgat5*^WT^ mice (Figure 1(o,p)). No significant differences were observed in the expression of occludin between *Mgat5*^WT^ and *Mgat5*^−/−^ mice (Supplementary Figure S1L). Altogether, these results demonstrate the functional impact of changes in the gut mucosa *N*-glycome composition in triggering alterations of gut microbiome alongside with perturbations of the intestinal barrier integrity.

### Sharing of the intestinal microbiota between Mgat5^−/−^ and Mgat5^WT^ mice impacts colitis susceptibility

We then sought to explore the role of the dysbiotic microbiota imposed by changes in host glycocalyx, in defining susceptibility to intestinal inflammation. To explore this, *Mgat5*^−/−^ and *Mgat5*^WT^ mice were co-housed (in the same cage), aiming to promote the sharing of their microbiota in a glycoengineered-dependent environment. Upon co-housing, colitis was induced with DSS (Figure 2(a)). Interestingly, co-housed *Mgat5*^−/−^ mice (CH-*Mgat5*^−/−^) developed a less severe disease, as indicated by lower DAI and area under the curve (AUC) when compared with single-housed *Mgat5*^−/−^ (SH-*Mgat5*^−/−^) (Figure 2(b,c)). Remarkably, co-housed *Mgat5*^WT^ (CH-*Mgat5*^WT^) exhibited a worse disease course (Figure 2(b,c)), which supports the relevance of host glycosylation in dictating the susceptibility to intestinal inflammation in a process mediated by microbiota alterations.

Gut microbiota composition analysis upon co-housing further revealed a clear gain in terms of increased abundance and diversity in CH-*Mgat5*^−/−^ comparing with SH-*Mgat5*^−/−^ (Figure 2(d,e)), which is in line with the milder disease symptoms. Indeed, upon co-housing, colitis-susceptible *Mgat5*^−/−^ mice exhibited a pronounced modulation of gut microbiota, mainly characterized by the enrichment of Firmicutes bacteria, the downregulation of Proteobacteria and Actinobacteriota phyla, as well as an enrichment in species belonging to the Turicibacter genus (Figure 2(f–h)).

These results demonstrate the clear impact of the intestinal microbiota profile induced by mucosa *N*-glycosylation alterations (modeled in *Mgat5*^*-/-*^ mice), in defining susceptibility to intestinal inflammation, putting glycans at the frontiers of host-microbiota interactions.

### *Impaired β1,6-GlcNAc complex branched* N-*glycosylation leads to adownregulation of ILC3-IL-22 mediated immune response in the gut*

**Figure 3.**
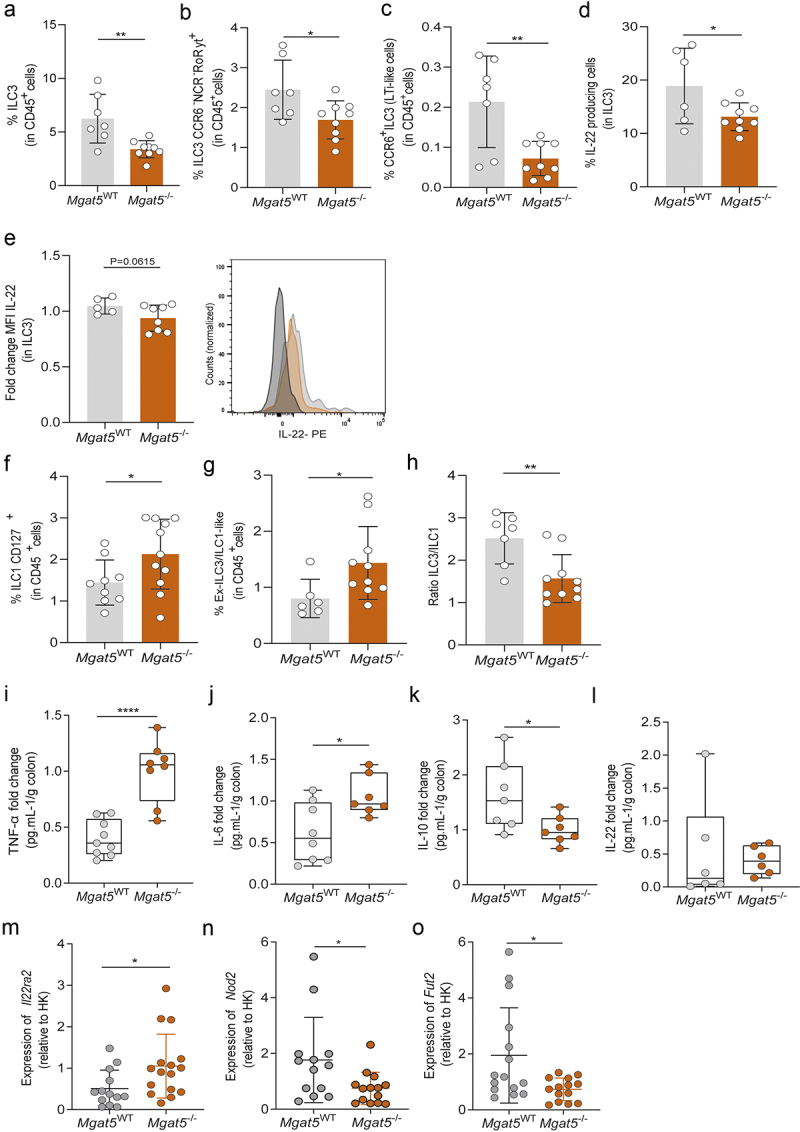
Deficiency in mucosa β1,6-GlcNAc complex branched *N*-glycosylation leads to an impaired ILC3-IL-22-mediated immune response. Frequency of (a) ILC3, (b) CCR6^−^NCR^−^RoRγt^+^ ILC3, and (c) CCR6^+^ILC3 (LTi-like) subsets in CD45^+^ cell population in *Mgat5*^WT^ and *Mgat5*^*-/-*^ mice, at steady state. (d) Frequency of IL-22–producing ILC3 and (e) mean fluorescence intensity (MFI) of intracellular IL-22 in ILC3 of *Mgat5*^WT^ and *Mgat5*^*-/-*^ mice at steady state. Frequency of (f) CD127^+^ILC1, and (g) ex-ILC3/ILC1-like cell subsets in CD45^+^ cell population in *Mgat5*^WT^ and *Mgat5*^*-/-*^ mice, at steady state. (h) Ratio between the frequencies of ILC3 and CD127^+^ILC1 in *Mgat5*^WT^ and *Mgat5*^*-/-*^ mice at steady state. (i-l) Cytokine concentrations in supernatants of colonic explants cultured for 24 hours. The concentrations are normalized to tissue weight. (m-o) The mRNA expression levels at steady state of genes encoding for (m) Il22bp, (n) Nod2 and (o) Fut2 in the colonic tissue measured by RT-qPCR. Expression of target gene mRNA was calculated based on housekeeping gene (*Gapdh*). mRNA expression levels were normalized for the average of mRNA levels of *Mgat5*^−/−^ mice. (a) *n* = 7–9 per group. (b, c, f, g and h) *n* = 6–11 per group. (d and e) *n* = 6–9 per group. (i-l) *n* = 6–8 per group. (m-o) *n* = 13–16 per group. Each datapoint represents an individual animal. Data is represented as mean ± SD. *p < 0.05; **p < 0.01; ****p < 0.0001 using an unpaired two-tailed Student’s *t*-test or Mann–Whitney test.

Taking into consideration the impact of gut mucosa *N*-glycome in shaping microbiota composition associated with severity to colitis, we then investigated their mechanistic effect in the regulation of intestinal inflammatory pathways. An extensive characterization of the different immune subsets in intestinal mucosa was performed at baseline (Supplementary Figure S2 and Figure 3) and after colitis induction (Supplementary Figure S3), comparing *Mgat5*^WT^ and *Mgat5*^−/−^ mice. No significant alterations were observed in the frequency of total T cells (CD3^+^), Th17 cells, regulatory T cells (Treg), B cells or macrophages at baseline (Supplementary Figures S2B-F). However, a remarkable downregulation in type 3 innate lymphoid cells (ILC3) was found in *Mgat5*^−/−^ mice, at baseline, in comparison with *Mgat5*^WT^ mice (Figure 3(a)). This significantly decreased expression of ILC3 was maintained upon disease induction (Supplementary Figure S3A). Specifically, and among the sub-populations of ILC3, we observed that the deficiency in mucosa complex branched *N*-glycans appears to selectively impact the downregulation of the CCR6^−^NCR^−^RORγt^+^ and CCR6^+^ILC3 (LTi-like cells) sub-populations at baseline (Figure 3(b,c)), contrary to NCR^+^RORγt^+^ ILC3 (Supplementary Figure S2G). This impact in innate immune response was also observed through the upregulation of dendritic cells in *Mgat5*^−/−^ mice at baseline in comparison with *Mgat5*^WT^ (Supplementary Figure S2H).

After disease induction, as expected, alongside a decrease in ILC3 expression (as well as CCR6^+^ILC3 (LTi-like cells) sub-population), (Supplementary Figures S3A,B), we also observed an increased Th17 response (Supplementary Figure S3C), with no alterations found in other immune sub-populations (Supplementary Figures S3D-G). Furthermore, a decrease in the production of IL-22 by ILC3 cells in *Mgat5*^−/−^ mice (Figures 3(d,e)) was observed and maintained upon colitis induction (Supplementary Figures S3H,I). This impact in IL-22 production in *Mgat5*^−/−^ mice were found to be specific for the ILC3 population since no differences were observed in IL-22 produced by Th17 cells both at baseline (Supplementary Figures S2I,J) and upon disease induction (Supplementary Figures S3J,K). Additionally, after DSS-induced colitis, but not at baseline (Suplementary Figures S2K,L), *Mgat5*^−/−^ mice displayed reduced levels of IL-22 production by ILC3 NCR^+^RORγt^+^ (Supplementary Figures S3L,M).

ILCs are highly plastic immune cells, that are able to adapt their phenotype and function depending on the environmental cues, such as dietary signals and microbial counterparts.^53^ The balance between ILC3 and ILC1 immune populations is known to play a key role in gut homeostasis, whereas disruption of this balance has been described as contributing to intestinal inflammation, such as IBD.^54^ However, whether changes in host glycocalyx may affect this ILC3/ILC1 immune balance remain unknown. Our results reveal the direct interplay between mucosa *N*-glycosylation and ILCs activity and function (Figure 3). In addition, we also showed that *Mgat5*^−/−^ mice displayed a clear trans-differentiation of ILCs population toward an ILC1 phenotype (Figure 3(f)), as observed by the increase in ex-ILC3/ILC1-like cells (Figure 3(g) and Supplementary Figures S2M-P). This is also demonstrated by the reduced ILC3/ILC1 ratio in *Mgat5*^−/−^ mice (Figure 3(h)). A similar profile was also found in *Mgat5*^−/−^ mice after DSS-induced colitis (Supplementary Figures S3N,O). These results demonstrate, for the first time, the direct impact of changes in mucosa *N*-glycosylation in the regulation of ILC3 module in the trans-differentiation toward an ILC1-mediated proinflammatory phenotype. This result is in line with previous observations reporting ILC3-to-ILC1 transition in inflamed mucosal biopsies from CD patients associated with anti-GM-CSF autoantibodies against glycosylation.^55^ This proinflammatory environment imposed by the ILC3/ILC1 plasticity, triggered upon alterations in gut mucosa glycome, was further supported by the increased production of TNFα and IL-6 proinflammatory cytokines in colonic explants from *Mgat5*^−/−^ mice at baseline (Figure 3(i,j)) and upon colitis induction (Supplementary Figures S3P,Q), and by the decreased levels of anti-inflammatory IL-10 cytokine at baseline (Figure 3(k)). After DSS-induced colitis, no major differences in terms of IL-10 secretion were observed comparing *Mgat5*^−/−^ and *Mgat5*^WT^ mice (Supplementary Figure S3R). In the same line, no alterations were found to IL-22 production in colonic explants at baseline and upon colitis induction (Figure 3l and Supplementary Figure S3S). Nevertheless, the impaired IL-22 signaling observed in *Mgat5*^−/−^ mice was also supported by the significant increased expression of *Il22ra2*, the gene encoding to IL22 binding protein (IL-22BP) (Figure 3(m)), a cytosolic protein involved in IL-22 blockade.^56,57^

IL-22 signaling is known to induce the production of nucleotide oligomerization domain-containing protein 2 (NOD2), which is related to the regulation of innate immune response and microbial recognition.^58^ Furthermore, *NOD2* is one of the most widely implicated genes in the etiology of Crohn’s disease.^59^ We here demonstrated that *Mgat5*^−/−^ mice also exhibited a significant downregulation of *Nod2* (Figure 3(n)), reinforcing the negative impact of the impaired IL-22 signaling (either by decreased IL-22-derived ILC3 or increased *Il22ra2)* in the homeostatic gut environment. In addition, and in line with previous evidence suggesting that IL-22 produced by ILC3 impacts epithelial cell glycosylation by regulating the expression of fucosyltransferase 2 (Fut2),^60^ we also observed that *Fut*2 expression was decreased in *Mgat5*^−/−^ mice (Figure 3(o)).

Taking together, these results demonstrate the clear impact of the deficiency in the β1,6-GlcNAc complex branched *N*-glycans (associated with dysbiosis) in the impairment of ILC3-IL-22 immune response that culminate in *Nod2* downregulation, that together converge in the activation of inflammatory cues promoting health to intestinal inflammation transition.

### Prophylactic remodeling of mucosa glycosylation through metabolic supplementation with glycans controls immune response by repairing ILC3-IL22 homeostatic immune pathway.

**Figure 4.**
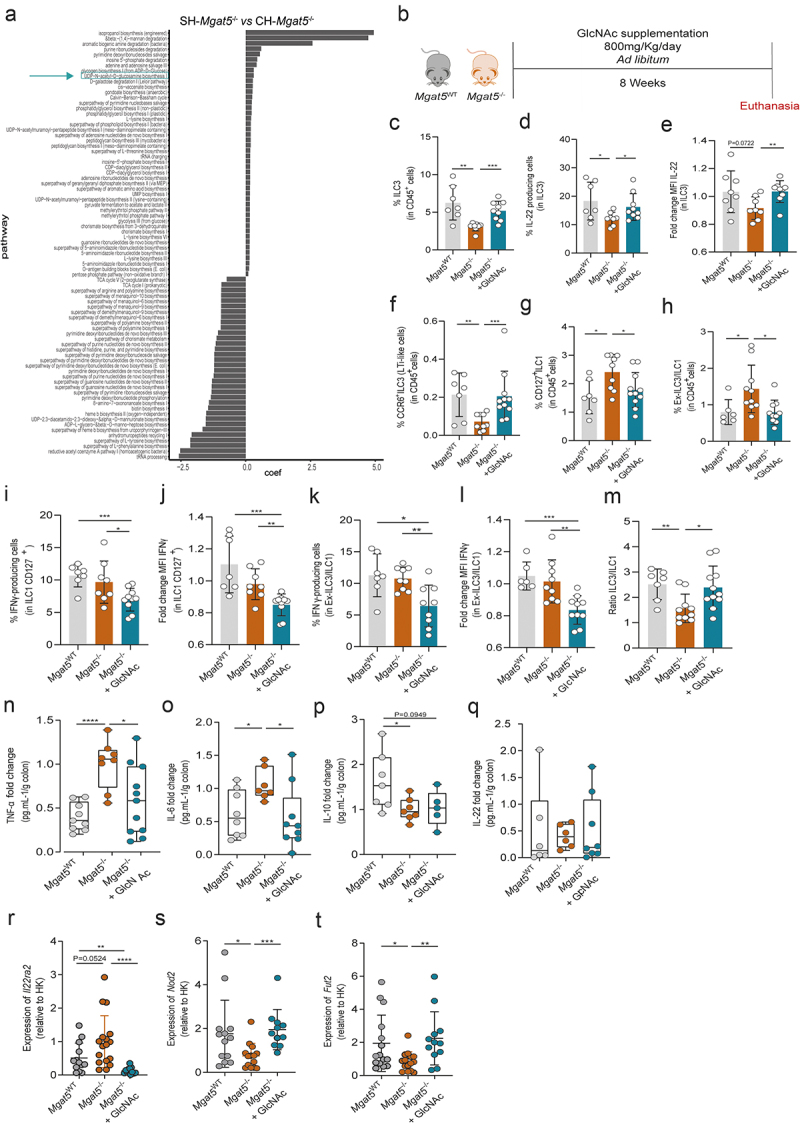
Prophylactic GlcNAc supplementation modulates immune response of *Mgat5*^−/−^ mice rescuing ILC3-IL22- homeostatic axis. (a) Unstratified pathway output of Picrust2 based on 16S rRNA sequencing of single- and co-housed (SH and CH) *Mgat5*^*-/-*^ mice. (b) Schematic representation of 800 mg/Kg/day *N*-acetylglucosamine (GlcNac) supplementation, *ad libitum*, of *Mgat5*^−/−^ mice for 8 weeks. (c) Frequency of ILC3 in CD45^*+*^ population in *Mgat5*^WT^, *Mgat5*^*-/-*^, and GlcNAc-supplemented *Mgat5*^*-/-*^ mice. (d) Frequency of IL22– producing ILC3 and (e) mean fluorescence intensity (MFI) of intracellular IL-22 in ILC3, in *Mgat5*^WT^, *Mgat5*^*-/-*^, and GlcNAc-supplemented *Mgat5*^*-/-*^ mice. (f-h) Frequency of (f) CCR6^+^ILC3 (LTi-like), (g) CD127^+^ILC1, and (h) ex-ILC3/ILC1-like cells in CD45^+^ cell population in *Mgat5*^WT^, *Mgat5*^*-/-*^, and GlcNAc-supplemented *Mgat5*^*-/-*^ mice. (i) Frequency of IFNγ–producing CD127^+^ILC1 and (j) mean fluorescence intensity (MFI) of intracellular IFNγ in CD127^+^ILC1, in *Mgat5*^WT^, *Mgat5*^*-/-*^, and GlcNAc-supplemented *Mgat5*^*-/-*^ mice. MFI were normalized for the average of *Mgat5*^−/−^ mice MFI. (k) Frequency of IFNγ–producing ex-ILC3/ILC1-like cells and (l) mean fluorescence intensity (MFI) of intracellular IFNγ in ex-ILC3/ILC1-like cells, in *Mgat5*^WT^, *Mgat5*^*-/-*^, and GlcNAc-supplemented *Mgat5*^*-/-*^ mice. MFI were normalized for the average of *Mgat5*^−/−^ mice MFI. (m) Ratio between the frequencies of ILC3 and CD127^+^ ILC1 in *Mgat5*^WT^, *Mgat5*^*-/-*^, and GlcNAc-supplemented *Mgat5*^*-/-*^ mice. (n-q) Cytokine concentrations in culture supernatants of colonic explants. The concentrations are normalized to tissue weight. (r-t) The mRNA expression levels at steady state of genes encoding for (r) Il22bp, (s) Nod2 and (t) Fut2 in the colonic tissue measured by RT-qPCR. Expression of target gene mRNA was calculated based on housekeeping gene (*Gapdh*). mRNA expression levels were normalized for the average of mRNA levels of *Mgat5*^−/−^ mice. (c-e) *n* = 8–10 per group. (f-h) *n* = 9–11 per group. (i-m) *n* = 8–11 per group (n, q) *n* = 6–11 per group. (r-t) *n* = 11–17 per group. Each datapoint represents an individual animal. Data is represented as mean ± SD. *p < 0.05; **p < 0.01; ***p < 0.001; using an unpaired two-tailed Student’s t-test or Mann–Whitney test.

To gain mechanistic insights into the microbial-derived pathways underlying the protective colitis phenotype that was observed in CH-*Mgat5*^−/−^ mice, an unstratified pathway output of Picrust2 analysis based on 16S rRNA data of SH-*Mgat5*^−/−^ and CH-*Mgat5*^−/−^ mice was performed (Figure 4(a)). The results demonstrated that CH-*Mgat5*^−/−^ mice exhibited the clear upregulation of the general microbial-associated traits involved in glycosylation biosynthetic pathways, particularly the UDP-*N*-acetylglucosamine biosynthesis pathway (Figure 4(a)). In fact, UDP-GlcNAc is the preferential substrate for the activity of GnT-V that results in the synthesis of β1,6-GlcNAc complex branched *N*-glycans.^30^ The upregulation of this selected microbial-derived trait involved in UDP-*N*-acetylglucosamine biosynthesis pathway upon mouse co-housing, associated with prevention of inflammation and control of dysbiosis (Figure 2), highlights the potential regulatory/protective involvement of complex branched *N*-glycans in host-microbial relationship associated with gut homeostasis.

To further test this hypothesis, we metabolically supplemented colitis-susceptible *Mgat5*^−/−^ mice with GlcNAc before disease induction (Figure 4(b)). This was specifically aimed at testing the prophylactic effect of the glycosylation remodeling in preventing inflammation. Notably, we observed that the supplementation of *Mgat5*^−/−^ mice with GlcNAc for 8 weeks was able to reestablish the levels of ILC3 (Figure 4(c)) and, importantly, of the homeostatic cytokine IL-22 produced by ILC3, at similar levels of *Mgat5*^WT^ (Figures 4(d,e)) at steady state. The prophylactic supplementation with GlcNAc also led to the upregulation of CCR6^+^ILC3 (LTi-like cells) and CCR6^−^NCR^−^RORγt^+^ILC3 sub-populations (Figure 4(f) and Supplementary Figure S4A). Notably, GlcNAc supplementation was also able to significantly reduce the frequency of ILC1 (Figure 4(g)) and ex-ILC3/ILC1 like (Figure 4(h)), as well as decrease IFNγ produced by these cells in the colon (Figures 4(i,l)). Indeed, GlcNAc supplementation significantly increased the ILC3/ILC1 ratio in *Mgat5*^−/−^ mice at similar levels of *Mgat5*^WT^ (Figure 4(m)). No differences were found in ILC3 NCR^+^RORγt^+^ and Treg cells (Supplementary Figures S4B and 4C). Nevertheless, we observed a decrease in the frequency of Th17 cells after glycan supplementation, with no impact on its IL-22 production (Supplementary Figures S4D-F). Accordingly, the effect of GlcNAc supplementation was further demonstrated by the reduced levels of proinflammatory cytokines IL-6 and TNFα in colonic supernatants, comparing treated *versus* non-treated mice, at baseline (Figure 4(n,o)). No alterations were found for IL-10 and IL-22 production (Figures 4(p,q)). Remarkably, we showed that GlcNAc treatment was able to reduce the expression of *IL22ra2* compared to both *Mgat5*^−/−^ and *Mgat5*^WT^ (Figure 4(r)). Importantly, the prophylactic effect of glycan supplementation was also observed in the rescue of the expression levels of *Nod2* and *Fut2* (Figures 4(s,t)). GlcNAc supplementation was also tested in *Mgat5*^WT^ mice, and no major alterations were found between *Mgat5*^WT^ treated and non-treated with GlcNAc in terms of ILC3 abundance and ILC3-derived IL22 production (Supplementary Figure S4G-I); however, GlcNAc supplementation significantly decreased IL-22 production in Th17 cells (Supplementary Figures S4J-L). Additionally, *Mgat5*^WT^ treated mice also display a trend decrease in IL-10 levels in colonic explants (Supplementary Figure S4M) with no major significant differences observed for the production of TNFα, IL-6, and IL-22 (Supplementary Figures S4N-P). No changes were observed in the expression of *Il22ra*, *Nod2* and *Fut2* in *Mgat5*^WT^ of GlcNAc treated mice (Supplementary Figures S4Q-S).

Overall, these data demonstrate the biological effect of prophylactically remodeling mucosa glycosylation as a promising strategy to control a key homeostatic intestinal module mediated by ILC3 and IL-22 dynamics.

### GlcNAc prophylactic supplementation is able to prevent severe colitis

**Figure 5.**
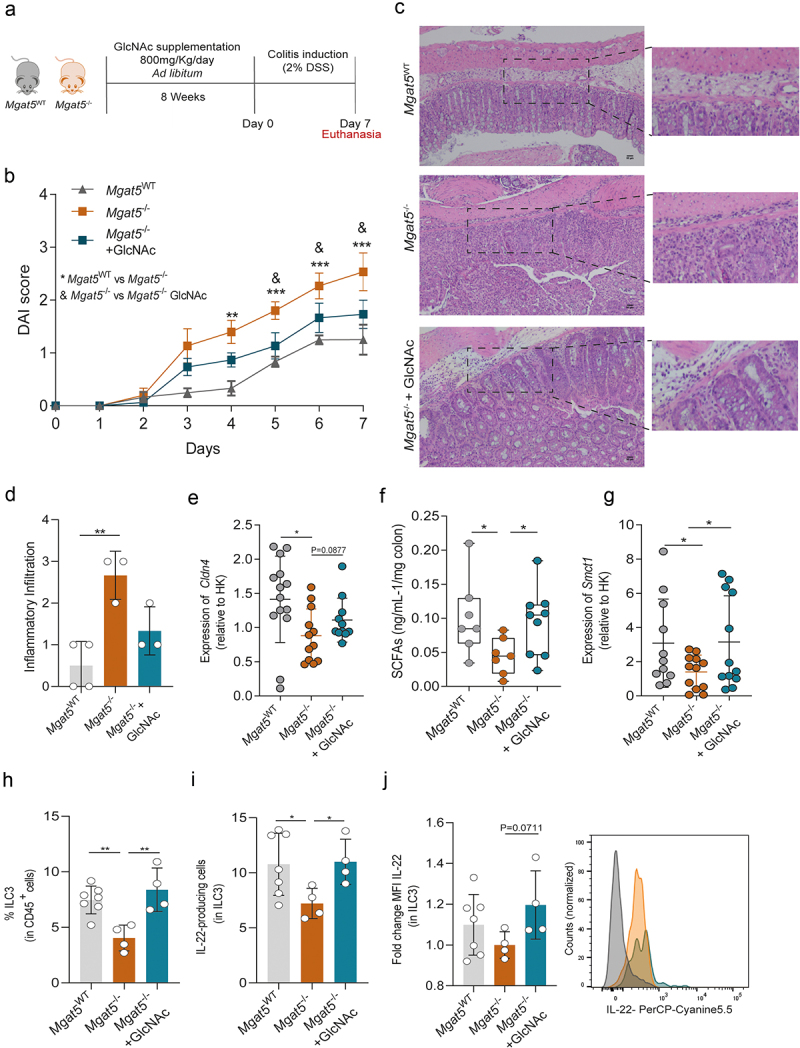
Glycan supplementation has a protective effect against colitis development and restores the levels of SCFAs. (a) Schematic representation of 800 mg/Kg/day *N*-acetylglucosamine (GlcNAc) supplementation of *Mgat5*^−/−^ mice for 8 weeks followed by 2% DSS treatment for 7 days. (b) Disease activity score (DAI) of *Mgat5*^WT^, *Mgat5*^*-/-*^, and GlcNAc-supplemented *Mgat5*^*-/-*^ mice upon DSS-induced colitis. (c and d) Histological analysis of hematoxylin & eosin staining and qualitative scores of intestinal immune infiltration of colon sections from *Mgat5*^WT^, *Mgat5*^*-/-*^, and GlcNAc-supplemented *Mgat5*^*-/-*^ mice upon DSS-induced colitis. There is no statistical significance between *Mgat5*^*-/-*^ and GlcNAc-supplemented *Mgat5*^−*/*-^ mice. Zoomed images highlight immune infiltrate. Scale bar = 50 μm. (e) The mRNA expression levels at steady state of *Cldn4* in *Mgat5*^WT^, *Mgat5*^*-/-*^, and GlcNAc-supplemented *Mgat5*^*-/-*^ mice at steady state. Expression of target gene mRRNA was calculated based on housekeeping gene (*Gapdh*). mRNA expression levels were normalized for the average of mRNA levels of *Mgat5*^*-/-*^ mice. (f) Quantification of SCFAs measured by ELISA in the colon of *Mgat5*^WT^, *Mgat5*^*-/-*^, and GlcNAc-supplemented *Mgat5*^*-/-*^ mice at steady state. (g) The mRNA expression levels at steady state of SCFAs transporter *Smct1* in *Mgat5*^WT^, *Mgat5*^*-/-*^, and GlcNAc-supplemented *Mgat5*^*-/-*^ mice at steady state. Expression of target gene mRNA was calculated based on housekeeping gene (*Gapdh*). mRNA expression levels were normalized for the average of mRNA levels of *Mgat5*^*-/-*^ mice. (h) Frequency of ILC3 in CD45^+^ population in *Mgat5*^WT^, *Mgat5*^*-/-*^, and GlcNAc-supplemented *Mgat5*^*-/-*^ mice upon DSS-induced colitis. (i) Frequency of IL22–producing ILC3 and (j) mean fluorescence intensity (MFI) of intracellular IL-22 in ILC3, in *Mgat5*^WT^, *Mgat5*^*-/-*^, and GlcNAc-supplemented *Mgat5*^*-/-*^ mice upon DSS-induced colitis. (b) *n* = 5 per group. (d) *n* = 3–4 per group. (e) *n* = 11–14 per group; (f) *n* = 7–9 per group. (g) *n* = 11–12 per group. (h-j) *n* = 4–7 per group. Each datapoint represents an individual animal. Data is represented as mean ± SD. *p < 0.05; **p < 0.01; using an unpaired two-tailed Student’s t-test or Mann–Whitney test.

Finally, we tested whether the prophylactic effect of GlcNAc supplementation is effective in preventing disease development. Thus, after 8 weeks of glycan supplementation, colitis was induced by DSS (Figure 5(a)). Remarkably, prophylactic glycan supplementation significantly reduced disease susceptibility and severity in comparison with non-treated *Mgat5*^−/−^ mice (Figure 5(b)). Histopathological analysis of colon sections, after DSS-colitis induction, revealed that treated animals showed a reduced inflammatory infiltrate when compared with non-supplemented mice (Figures 5(c,d)). To understand if the decreased susceptibility of supplemented mice was also due to a remodeling of the intestinal epithelial barrier, expression of claudin-encoding genes and intestinal permeability were assessed prior colitis induction. No alterations were found in intestinal permeability or in claudin-2 expression comparing non-treated and glycan-treated *Mgat5*^*-/-*^ mice (Supplementary Figures S5A,B). Nevertheless, a tendency to an upregulation of claudin-4 was observed (Figure 5(e)). GlcNAc supplementation in *Mgat5*^*-/-*^ mice did not display major differences relative to non-treated *Mgat5*^*-/-*^ mice in terms of microbiota, with only two mice exhibiting a trend to a divergent microbiota composition, closer to *Mgat5*^*WT*^ mice profile (Supplementary Figure S5F). This preliminary result suggests the need for longer periods of GlcNAc supplementation to see effects in the gut microbiota diversity and richness. However, and consistent with a protective phenotype imposed by supplementation with glycans, SCFAs levels were markedly increased upon glycan-supplemented *Mgat5*^−/−^ mice (Figure 5(f)), which is in accordance with increased levels of *Smct1* transporter (Figure 5(g)). No differences were observed for the Mct1 transporter and receptors (Supplementary Figures S5C-E). Importantly, upon DSS-induced colitis, *Mgat5*^*-/-*^ mice treated with GlcNAc exhibited enhanced ILC3 levels concomitantly with an increase in IL-22 production (Figures 5(h,j)).

Overall, these results pinpoint the immunomodulatory properties of GlcNAc supplementation in a colitis-susceptible mouse model. This is supported by the ability of glycans in shaping a tolerogenic gut immune profile by activating the protective ILC3-IL-22 module, thereby preventing health-to-intestinal disease transition.

### *Impaired mucosa* N-*glycosylation occurs concomitantly with decreased frequency of intestinal ILC3 in human IBD patients*

**Figure 6.**
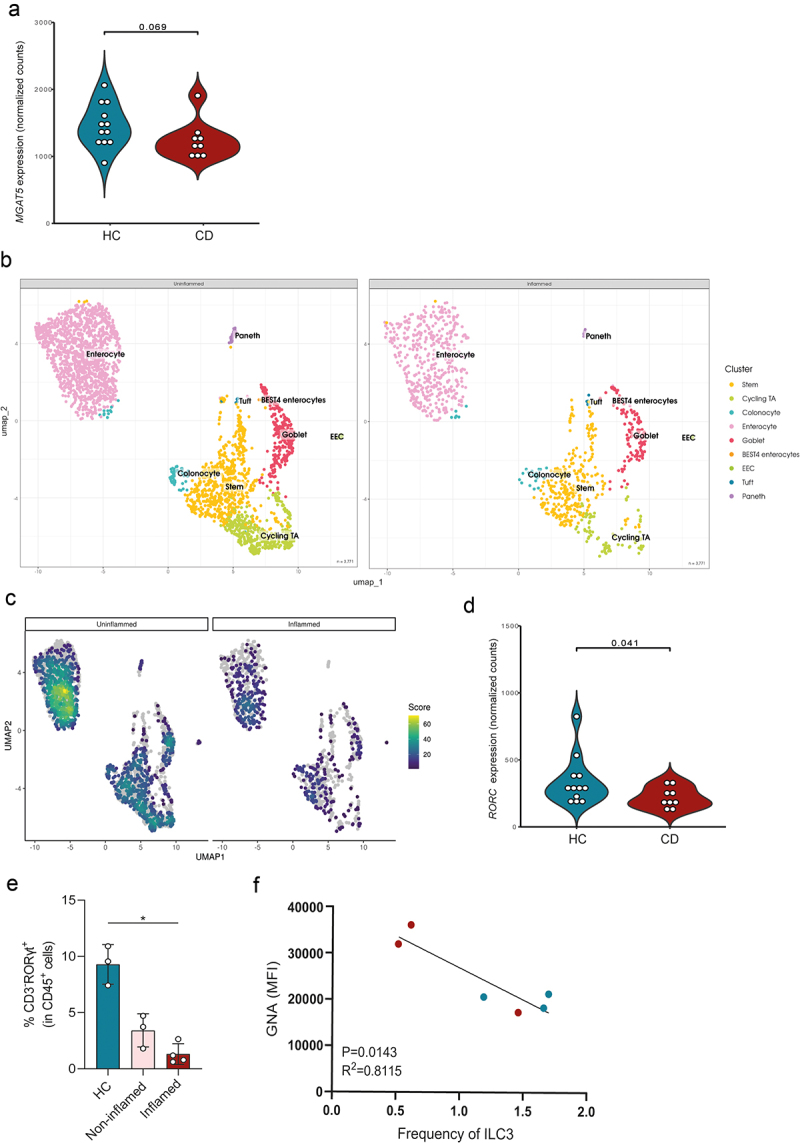
Impaired *N*-glycan-related glycogene expression in IBD patients associates with reduced frequency of intestinal mucosal ILC3. (a) The gene expression of *MGAT5* glycogene in the inflamed colonic tissue from CD patients and healthy controls. (b) Representation of epithelial cell clustering. Cell-type annotation was performed based on the expression profile of marker genes from published work on human intestinal tract. EPCAM-positive cells were divided into stem cells (*LGR5, ASCL2, SMOC2, RGMB, OLFM4*), Paneth (*DEFA5, DEFA6, REG3A*), cycling transit-amplifying (*MKI67, TOP2A, PCNA*), goblet cells (*CLCA1, SPDEF, FCGBP, ZG16, MUC2*), BEST4 enterocytes (*BEST4, OTOP2, CA7*), enterocytes (*RBP2, ANPEP, FABP2*) and colonocytes (*CA2, SLC26A2, FABP1*), enteroendocrine cells (*EEC; CHGA, CHGB, NEUROD1*), tuft cells (*POU2F3, LRMP, TRPM5*). (c) Gene signature of a set of GlcNAc transferases participating in the branching *N*-glycosylation pathway (*MGAT5, MGAT5B, MGAT4D, MGAT4C, MGAT4B, MGAT4A, MGAT3, MGAT1*) on epithelial cells. (d) The gene expression of *RORC* in the inflamed colonic tissue from CD patients and healthy controls. (e) Frequency of ILC3 (CD3^−^RORγt^+^) in CD45^+^ population in inflamed and non-inflamed mucosal tissue from IBD patients and healthy controls. (f) The coefficient correlation between frequency of ILC3 and GNA expression in inflamed mucosal tissue from IBD patients (red dots) and healthy controls (blue dots). (a and d) *n* = 9–12 per group. Data were derived from dataset published by Häsler et al.^46^ (e) *n* = 3–4 per group. Data are represented as mean ± SD *p < 0.05; using a one-way ANOVA test. (f) *n* = 3 per group; Pearson correlation test.

To further validate the specific impact of an altered intestinal glycome in the regulation of ILC-mediated gut immunity in IBD immunopathogenesis, we investigated whether changes in gut mucosa glycosylation may be linked to alterations in ILC3 module in human IBD. To do so, two publicly available databases (GSE134809, Martin et al.^44^; and Häsler et al.^46^) were analyzed. Based on the study of Häsler et al.,^46^ we found that the expression of *MGAT5* glycogene, involved in the production of complex branched *N*-glycans, appears to be impaired in inflamed colonic tissue of CD patients when compared to the controls (Figure 6(a)). Complementary, our analysis based on the study of Martin et al.^44^ further showed a reduced expression of a set of glycogenes participating in the branching *N*-glycosylation pathway, including *MGAT5* glycogene, in epithelial cells from inflamed CD biopsies (Figure 6(b,c)). Moreover, a downregulation of *MGAT5* glycogene in epithelial stem cells (Supplementary Figure S6A) was observed in inflamed CD biopsies when compared with non-inflamed samples. Interestingly, we also demonstrated that the expression of the transcription factor *RORC* (associated with ILC3) was significantly downregulated in inflamed CD biopsies (Figure 6(d); study Häsler et al.^46^) and the levels of ILC3 also seem to be decreased in inflamed CD biopsies (Supplementary Figure S6B; study Martin et al.^44^). This evidence supporting the link between *MGAT5* glycogene and ILC3 modulation was further validated in clinical samples obtained from mucosal biopsies of CD and UC patients, as well as healthy individuals (controls) (Supplementary Table S1). Accordingly, the results showed that inflamed biopsies from IBD patients displayed a significant decreased frequency of ILC3 compared to healthy controls (Figure 6(e)). Concomitantly, the expression of less complex, mannose-enriched *N*-glycans (GNA binding) at the epithelial cells was inversely correlated with frequency of ILC3, suggesting that a deficiency in complex branched *N*-glycosylation and decreased frequency of gut ILC3 in IBD are intricately related (Figure 6(f)). Altogether, these findings support the association between loss of complex *N*-glycans (through downregulation of *MGAT5*) with impaired levels of ILC3 at intestinal mucosa, associated with IBD.

## Discussion

The loss of equilibrium between microbes and the intestinal immune system is one of the main drivers of the onset of IBD.^4,61,62^ In fact, recent evidence points toward altered host-microbiome interactions as one of the earliest events in the pathogenesis of intestinal inflammation.^28^

Protein glycosylation has emerged as a possible link between host microbiome and immune response. We and others have previously demonstrated the implications of glycans in gut immunity and in the immunopathogenesis of IBD,^28^ even years before IBD diagnosis.^21^ However, whether and how an altered host glycocalyx contribute to the loss of immune tolerance, by imposing alterations in microbiota composition (dysbiosis) and the activation of immune response, is a fundamental question that remains unanswered.

In this study, we demonstrated that alterations in host mucosa *N*-glycans are at the basis of the crosstalk between the gut microbiome and the host immune response associated with health to intestinal inflammation transition. We revealed that a deficiency in the expression of complex branched *N*-glycans leads to a clear dysbiosis, together with the perturbation of the gut permeability, that were associated with colitis severity. This dysbiotic phenotype was characterized by a significant reduction in protective bacteria from Firmicutes phylum, namely *Blautia, Ruminococcaceae NK4A214 group* and *Lachnoclostridium* genera, concomitantly with the overgrowth of selective pathobionts, such as families from the Actinobacteriota, Proteobacteria and Bacteroidota phyla, considered to be general glycan degraders.^63,64^ In turn, *Blautia, Ruminococcaceae NK4A214 group* and *Lachnoclostridium* genera are known for producing SCFAs, mainly propionate and butyrate, playing a significant role in strengthening the intestinal barrier, maintaining intestinal homeostasis, and preventing inflammation.^65^

The mucosa glycan switch, imposed by a deficiency in complex branched *N*-glycans and the abnormal exposure of less complex/mannose-enriched glycans alongside dysbiotic microbiota, was also found to compromise the intestinal barrier, as observed by changes in the expression of claudins and tight junctions. This impact of changes in mucosa glycome in gut permeability is in line with previous evidence revealing that increased intestinal permeability may predict later development of CD.^66^

We further demonstrated that changes in mucosa glycosylation not only led to dysbiosis, but directly perturb a key homeostatic module in the intestine mediated by ILC3 immune response. In fact, ILC3 are known to be a central player in orchestrating tissue and gut immune homeostasis at steady state.^67^ ILC3 control the immune response against pathogens and opportunistic commensals,^60,68–70^ representing the main source of protective IL-22 in the intestine.^71^ Diet and commensal microbiota-derived signals are described to regulate ILC3 recruitment and function in the intestine.^71–73^ Here we showed, for the first time, that a deficiency in complex branched *N*-glycans at gut mucosa results in a significant decrease of ILC3 and ILC3-producing IL-22 revealing mucosa glycans as a new source of ILCs regulators. This downregulation of ILC3, imposed by the deficiency in complex branched *N*-glycans, further promoted its plasticity toward an ILC1 phenotype. Specifically, we showed that the deficiency in mucosa complex branched *N*-glycans in *Mgat5*^−/−^ mice induced the downregulation of CCR6^+^ILC3 (LTi-like ILC3) and CCR6^−^NCR^−^RORγt^+^ cell subsets. LTi-like ILC3 were described to contribute towards gut homeostasis, tight junction expression and IL-22 production,^74–76^ being the prevailing ILC3 subset in colonic lamina propria of healthy mice.^77,78^ CCR6^−^NCR^−^ILC3 can transform into CCR6^−^NCR^+^ILC3 through the upregulation of T-bet and Notch.^79^ Our results suggest that the development of these ILC3 precursors may be impaired through changes in mucosa glycosylation associated with altered microbiota and respective signals. Furthermore, this effect of the mucosa glycan switch in the perturbation of ILC3 module is also in line with the impact observed in *Nod2* expression and in *Fut2*-mediated glycosylation. NOD2 is an intracellular pattern recognition receptor present in most intestinal immune and epithelial cells, and is known to recognize glycans, such as bacterial peptidoglycans, being involved in IBD immunopathogenesis.^59^ Accordingly, we also observed that the downregulation of IL-22-producing ILC3 imposed by glycan alterations was associated with decreased expression of *Fut2*-mediated fucosylation and with susceptibility to inflammation, despite we found only a slight decrease in UEA-I binding. This is in accordance with previous evidence suggesting that IL-22 produced by ILC3 can act as a modulator of intestinal epithelial fucosylation by *Fut2* expression associated with homeostasis.^60^

Overall, we demonstrated that deficiency in complex branched *N*-glycans at gut mucosa surface imposes a clear pro-inflammatory microenvironment, driven by ILC3/ILC1 module with a concomitant overexpression of other proinflammatory cytokines, such as TNFα and IL-6 and the downregulation of the anti-inflammatory cytokine IL-10. This further supports the biological relevance of glycans and of mucosal *MGAT5* downregulation in the overall reprograming of the pro-inflammatory environment in the intestine.^8,21^ Indeed, pro-inflammatory cytokines, such as TNFα, are recognized to both increase the permeability of the intestinal epithelial barrier and to impair the expression of SCFA transporters and receptors, resulting in the disruption of essential host-commensal mutualistic pathways.^80–82^ This goes in line with the impact of a deficient complex branched *N*-glycosylation in TNFα production.

Importantly from the clinical point of view, the association between an altered mucosal glycosylation and ILC3 module was validated in human IBD clinical samples, revealing that impaired complex branched *N*-glycosylation in gut epithelial cells associates with deficient intestinal ILC3, which could reinforce the clinical impact of host mucosa glycome and ILC3 immune response associated with IBD. Nevertheless, further studies are required to validate this association in a clinical setting.

Taken together, we have here disclosed, at a mechanistic level, the biological effects of changing the intestinal *N*-glycome composition in triggering gut permeability and dysbiosis, the downregulation of protective ILC3/IL22 axis associated with decreased *Nod2* expression, as well as the activation of immune pathways that culminate in intestinal inflammation.

Intestinal commensal-derived signals from the microbiota are pivotal in maintaining gut homeostasis through the regulation of the differentiation and proliferation of both intestinal epithelial and immune cells.^83^, Among the ILC population, ILC3s have been found to be tightly regulated by commensal microbial signals, while ILC1s are highly promoted under proinflammatory environments.^84^, ILC3 can be directly regulated by SCFAs produced by microbiota, promoting ILC3 proliferation and IL-22 production.^72^ In accordance, ILC3 was found to express SCFAs receptors, such as *Gpr109a* and *Gpr43*,^72,85^ highlighting the relevance of microbiota-derived SCFA in modulating ILC3 immune response. In this study, we bring to light the importance of mucosa glycosylation in this triangle mediated by host glycome-microbiota and ILC3 module with impact in gut homeostasis and inflammation. We demonstrated that a deficiency in complex branched *N*-glycans alter the gut microbiota equilibrium, leading to dysbiosis and consequently to a reduction in SCFAs production with impact in ILC3-mediated immune response and the onset of intestinal inflammation.

The identification of a specific immunological axis controlled by mucosa *N*-glycans pave the way for exploring whether the prophylactic supplementation with glycans may enhance the biosynthesis of “tolerogenic” complex *N*-glycans and, in this way, prevent the health to intestinal inflammation transition. Remarkably, the prophylactic supplementation of glycoengineered colitis-susceptible mice with GlcNAc suppressed disease severity, by significantly ameliorating clinical symptoms. This clinical effect of GlcNAc supplementation was due to the restoration of the expression of the homeostatic ILC3-IL-22 module, with increased ILC3 frequency and blockade of ILC3-to-ILC1 transition. Moreover, the protective effect of GlcNAc supplementation was also highlighted by restoring protective levels of SCFAs and increased *Nod2* expression. This prophylactic effect of glycans supplementation in remodeling gut milieu associated with immunotolerance and gut homeostasis is promising, deserving further exploitation in pre-clinical and clinical settings.

**Figure 7.**
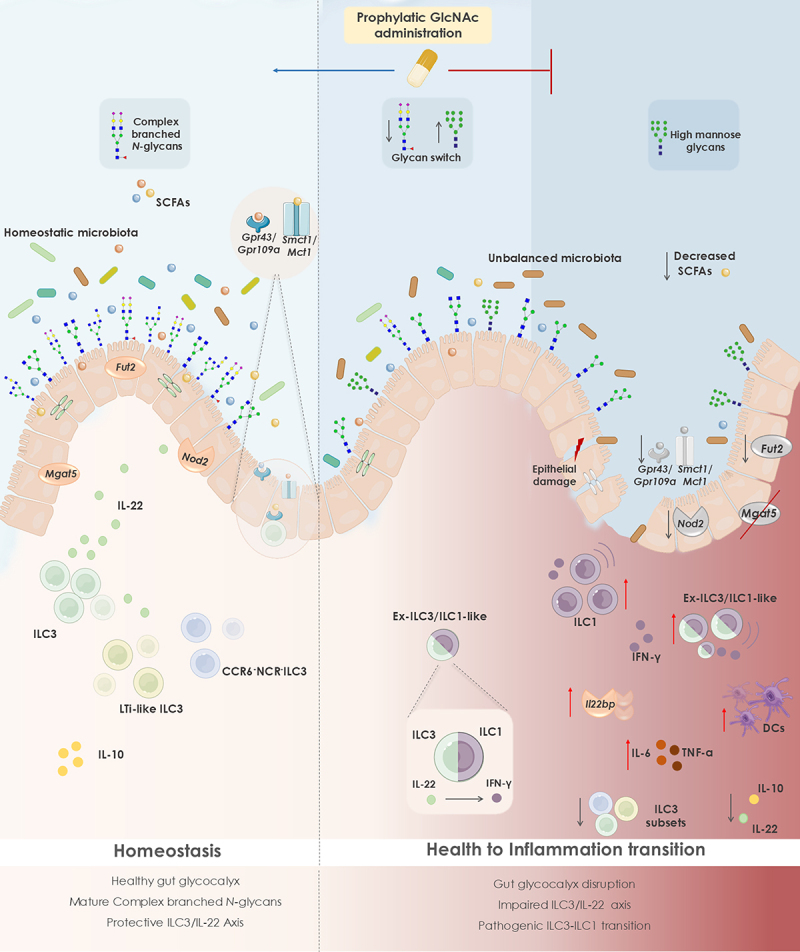
Changes in mucosa branched *N*-glycosylation impacts gut microbial balance, leading to an intestinal inflammatory response. In a healthy gut glycocalyx, epithelial branched *N*-glycans sustain commensal gut microbiota composition, contributing to the integrity of the epithelial barrier. ILC3 subsets, including LTi-like ILC3 and CCR6^−^NCR^−^ILC3, promote gut homeostasis through IL-22 release. Short-chain fatty acids (SCFAs) produced by commensal microbiota regulate mucosal homeostasis through activation of Gpr43/Gp109a signaling pathways or entering cells through Mct1 and Smct1 transport. Intestinal epithelial fucosylation (via *Fut2* expression) and microbiota recognition (through *Nod2* expression) also support the symbiotic relationship between microbes and the intestinal immune system in homeostasis. During health to intestinal inflammation transition, there is an altered host glycome profile, characterized by a deficiency in the mucosa expression of complex branched *N*-glycans and exposure of mannose-enriched glycans, perturbing mucosal integrity. This imbalanced glycoenvironment leads to a dysbiotic gut microbiota and deficient SCFAs release. Signaling and transport of SCFAs is also impaired. Concomitantly, this mucosa glycan switch hampers a protective immune response, through decreased frequency of ILC3 subsets, reduction in homeostatic cytokines (IL-22 and IL-10) and downregulation of *Nod2* and *Fut2* expression in the intestinal mucosa. An ILC3-ILC1 plasticity occurs imposed by the mucosa glycosylation alteration, leading to a pathogenic ILC1 proinflammatory phenotype, with increased TNFα and IL-6 production and expression of inhibitory IL-22 binding protein, which has the ability to block IL-22. The frequency of dendritic cells (DCs) is also increased upon deficiency in *Mgat5*-mediated branched *N*-glycans. Taken together, this results in a pro-inflammatory environment in the gut and a consequent shift from health to inflammation. Prophylactic supplementation with GlcNAc, a key metabolite in the hexosamine biosynthetic pathway, restores the expression of the protective ILC3-IL-22 module, suppressing health to intestinal inflammation transition. This ultimately highlights the beneficial impact of mucosa glycosylation remodeling through nutritional intervention in order to promote intestinal homeostasis.

Altogether, our findings unlock the potential beneficial effects of targeting mucosa glycocalyx as a promising strategy to preserve a healthy crosstalk between microbiome and gut immunity and to prevent IBD development (Figure 7). Accordingly, the evidence suggesting the clinical and biological impact of glycome alterations in the preclinical phase of CD^21,86^ brings to light the prominent impact of glycans and mucosa glycosylation as a putative trigger of health to intestinal inflammation transition with promising clinical applications for IBD prediction and prevention.

Overall, this study unveils a new mechanism in gut immunity tightly regulated by host mucosa *N*-glycans as master shapers of microbiome composition and ILC3/ILC1 immune regulation. The remodeling of mucosa glycome through glycan supplementation showed clear prophylactic effects in preserving gut homeostasis by promoting the activation of ILC3-IL-22 protective module. These results emphasize the impact of mucosa glycans in the crosstalk between gut microbiota and immune regulation, revealing the potential use of glycans on precision nutrition approaches and therapeutic interventions for preventing health to intestinal inflammation transition and for IBD prevention.

## Supplementary Material

Supplemental Material

## Data Availability

Data, reagents, materials and protocols are available from the corresponding author upon request.
