# Supplementary material for: Alterations in mucosa branched N-glycans lead to dysbiosis and downregulation of ILC3: a key driver of intestinal inflammation
Source: Gut Microbes. 2025 Feb 7;17(1):2461210. doi: 10.1080/19490976.2025.2461210 (PMC11810091; doi:10.1080/19490976.2025.2461210)
Supplement: Supplemental Material [file KGMI_A_2461210_SM2141.zip › SupplementaryMaterial_Rodrigues_et_al_15012025.docx]

**Supplementary Material for**

**Alterations in mucosa branched *N*-glycans lead to dysbiosis and downregulation of ILC3: a key driver of intestinal inflammation.**

Cláudia S. Rodrigues^1,2*^; Joana Gaifem^1*^; Márcia S. Pereira^1,2^; Maria Francisca Alves^1,2,3^; Mariana Silva^1,2^, Nuno Padrão^1,4^, Bruno Cavadas^1^, Catarina Moreira-Barbosa^5^, Inês Alves^1^; Ricardo Marcos-Pinto^2,6, 7^, Joana Torres^8,9,10^, Aonghus Lavelle^11^; Jean-Frederic Colombel^12^; Harry Sokol^11,13,14,15^; Salomé S. Pinho^1,2,4#^

^1^ Institute for Research and Innovation in Health (i3S), Immunology, Cancer & Glycomedicine Group, University of Porto, Porto, Portugal.

^2^ School of Medicine and Biomedical Sciences (ICBAS), University of Porto, Porto, Portugal.

^3^ Faculty of Sciences, University of Porto, Porto, Portugal.

^4^ Faculty of Medicine, University of Porto, Portugal

^5^ Hospital da Luz Learning Health, Luz Saúde, Lisbon, Portugal

^6^ Department of Gastroenterology, Centro Hospitalar do Porto, Porto, Portugal

^7^ Centro de Investigação em Tecnologias e Serviços de Saúde, University of Porto, Porto, Portugal.

^8^ Division of Gastroenterology, Hospital Beatriz Ângelo, Portugal

^9^ Faculty of Medicine, University of Lisbon, Lisbon, Portugal

^10^ Division of Gastroenterology, Hospital da Luz, Lisbon, Portugal

^11^ Sorbonne Université, INSERM, Centre de Recherche Saint-Antoine, CRSA, AP-HP, Saint-Antoine Hospital, Gastroenterology Department, 75012 Paris, France;

^12^Henry D. Janowitz Division of Gastroenterology, Department of Medicine, Icahn School of Medicine at Mount Sinai, New York, NY, USA.

^13^ Université Paris-Saclay, INRAE, AgroParisTech, Micalis Institute, 78350 Jouy-en-Josas, France;

^14^ AP-HP, Service de Gastroenterologie, Hôpital Saint Antoine, Paris, France;

^15^ Paris Center for Microbiome Medicine (PaCeMM) FHU, Paris, France.

^#^Corresponding author: Salomé Pinho

**Contact:** salomep@i3s.up.pt

*These authors contribute equally to this work

**Supplementary Table 1. Demographic and clinical characteristics of the samples from inaugural IBD and healthy controls.**

|  | **Patients with IBD**  **(UC; CD)** | **Healthy control individuals** |
| --- | --- | --- |
| **Number of individuals** | 5  (2; 3) | 3 |
| **Number of samples** | 8 | 3 |
| **Age^a^**  years; mean ± SD | 43.0 ± 16.2 | 79.0 ± 8.0 |
| **Sex**  n | Female 5  Male 0 | 2  1 |

^a^Age at sample collection

**Supplementary Table 2. Disease activity index used to evaluate mice disease severity.**

| **DAI** | **Weight loss** | **Stool consistency** | **Bleeding** |
| --- | --- | --- | --- |
| 0 | No loss | WF | No blood in the swab |
| 1 | 1-5% | WF/P | Traces of blood in the swab |
| 2 | 6-10% | P | Clear presence of blood in the swab |
| 3 | 11-18% | P/L | Red-stained, clear presence of blood in the swab |
| 4 | >18% | L | Blood in anus at naked eye |

WF – well-formed; P – pasty; L – liquid.

**Supplementary Table 3. Antibodies and lectins used to flow cytometry.**

| **Antibodies** | **Source** | **Identifier** |
| --- | --- | --- |
| APC/Fire™ 810 anti-mouse CD45 (clone 30-F11) | BioLegend | Cat#: 103173 |
| Brilliant Violet 605 anti-mouse CD3 (clone 17A2) | BioLegend | Cat#: 100237 |
| eFluor 506 Anti Hu/Mo CD45R (B220) (clone RA3-6B2) | eBioscience | Cat#: 69-0452-82 |
| eFluor 506 Anti-Mo CD11c (clone N418) | eBioscience | Cat#: 69-0114-80 |
| eFluor 506 Anti-Mo Ly-6G/Ly-6C (clone RB6-8C5) | eBioscience | Cat#: 69-5931-80 |
| eFluor 506 Anti-Mo TER-119 (TER-119) | eBioscience | Cat#: 69-5921-80 |
| eFluor 506 Anti-Mo CD11b (clone M1/70) | eBioscience | Cat#: 69-0112-80 |
| PE/Cyanine5 anti-mouse CD335 (NKp46) (clone 29A1.4) | BioLegend | Cat#: 137647 |
| BV786 Rat anti-mouse CD90.2 (clone 53-2.1) | BD | Cat#: 564365 |
| Brilliant Violet™ 711 Anti-Mo CD127 (clone A7R34) | eBioscience | Cat#: 407-1271-82 |
| eFluor™ 660 Anti-Mo CD196 (CCR6) (clone sirx6) | eBioscience | Cat#: 50-7196-82 |
| APC Anti-Hu/Mo T-bet (clone 4B10) | eBioscience | Cat#: 17-5825-80 |
| PE-Cyanine7 Anti-Mo ROR gamma (t) (clone B2D) | eBioscience | Cat#: 25-6981-82 |
| PE-CF594 Mouse anti-EOMES (clone X4-83) | BD | Cat#: 567167 |
| FITC anti-mouse IL-17 (clone TC11-8H4) | Life Technologies | Cat#: A15377 |
| PE anti-mouse IL-22 (clone Poly5164) | BioLegend | Cat#: 516404 |
| Brilliant Violet™ 421 Anti-Mo GM-CSF (clone: MP1-22E9) | BD | Cat#: 564747 |
| PerCP-Cyanine5.5 Anti-Mo IFN gamma (clone: XMG1.2) | eBioscience | Cat#: 45-7311-80 |
| FITC Anti-Mouse CD45 (clone: 30-F11) | eBioscience | Cat#: 53-1071-82 |
| PE-Cyanine5 Anti-Mi MHC class II (I-A/I-E) (clone: M5/114.15.2) | eBioscience | Cat#: 15-5321-81 |
| PE Anti-Mo CD24 (clone: M1/69) | eBioscience | Cat#: 12-0242-81 |
| APC Anti-Mo CD64 (clone: X54-5/7.1) | eBioscience | Cat#: 17-0641-80 |
| eFluor 450 Anti-Mo CD11c (clone: N418) | eBioscience | Cat#: 48-0114-82 |
| PE-Cyanine7 Anti-Mo CD45 (clone: 30-F11) | eBioscience | Cat#: 25-0451-82 |
| APC/Fire 810 Anti-Human CD45 (clone: HI30) | BioLegend | Cat#: 304076 |
| Brilliant Violet 605 Anti-Human CD3 (clone: OKT3) | BioLegend | Cat#: 317322 |
| Alexa Fluor 647 Anti-Human Rorγt (clone: Q21-559) | BD | Cat#: 563620 |
| FITC Anti-Human CD19 (clone: HIB19) | BioLegend | Cat#: 302206 |
| PE Streptavidin | BioLegend | Cat#: 405203 |
| PE Spark Blue 550 Streptavidin | BioLegend | Cat#: 405363 |
| LPHA (495/515) | Vector Laboratories | FL-1111 |
| SNA (Cy5) | Vector Laboratories | CL-1305 |
| GNA (495/515) | Vector Laboratories | FL-1241 |
| MAL-II (biotinylated) | Vector Laboratories | B-1265 |
| UEA-I (biotinylated) | Vector Laboratories | B-1065 |

**Supplementary Table 4. Mouse oligonucleotides used in quantitative real-time PCR.**

| **Primers** | **Forward Sequence** | **Reverse Sequence** |
| --- | --- | --- |
| ***Fut2*** | AGGCGGTTCAAATGTCCTCA | CGTTGTGCATGGATTCAGGG |
| ***Il22ra2*** | TCAGCAGCAAAGACAGAAGAAAC | GTGTCTCCAGCCCAACTCTCA |
| ***Nod2*** | CTGTCCAACAATGGCATC | GTTCCCTCGAAGCCAAACCT |
| ***Gpr43*** | AATTTCCTGGTGTGCTTTGG | ACCAGACCAACTTCTGGGTG |
| ***Gpr109a*** | ATGGCGAGGCATATCTGTGTAGCA | TCCTGCCTGAGCAGAACAAGATGA |
| ***Smct1*** | TTATGGGCGGTCGCAGTA | CAGAGGCCCACAAGGTTGACAT |
| ***Mct1*** | TGTTGTTGCAAATGGAGTGT | AAGTCGATAATTGATGCCCATGCCAA |
| ***Ocludin*** | GCTGTGATGTGTGTTGAGCT | GACGGTCTACCTGGAGGAAC |
| ***Cldn1*** | GGCTTCTCTGGGATGGATCG | CCCCAGCAGGATGCCAATTA |
| ***Cldn2*** | TTTTGGGGCTGTTAGGCACA | AGAATCCTGGCAGAACACGG |
| ***Cldn3*** | GTACAAGACGAGACGGCCAA | CGTAGTCCTTGCGGTCGTAG |
| ***Cldn4*** | ACGTCATCCGCGACTTCTAC | TTGTCGTTGCTACGAGGTGG |
| ***Gapdh*** | GAAGGTCGGTGTGAACGGAT | CTCGCTCCTGGAAGATGGTG |

**Supplementary Figures Legends**

**Supplementary Fig. 1. Reduction of branched *N*-glycans in mice promotes gut dysbiosis and intestinal permeability.** (A) Disease activity score (DAI) of *Mgat5*^WT^ and *Mgat5^-/-^*upon DSS-induced colitis. Data represents 4 independent experiments. (B) Schematic representation of the *N*-glycosylation pathway from high-mannose *N*-glycans to complex *N*-glycans. Respective monomers recognized by the different lectins (GNA, LPHA, MAL-II, SNA and UEA-I). (C) Gating strategy for epithelial cells. Living cells were gated based on FVD staining and doublets were excluded using an FSC-H/FSC-A gate. Epithelial cells: CD45^-^, Epcam^+^ and E-chaderin^+^. (D and E) Levels of (D) β1,6-GlcNAc complex branched *N*-glycans (LPHA), (E) mannose-enriched *N*-glycans (GNA) at steady state in epithelial cells (CD45^-^E-chaderin^+^Epcam^+^ cells) from *Mgat5^-/-^* mice and *Mgat5^WT^* controls. (F) Ratio between the MFI of LPHA and GNA in *Mgat5^-/-^* mice and *Mgat5^WT^* at steady state. (G-I) Levels of (G) α1,2-fucose residues (UEA-I), (H) terminally α2,6-sialylated glycan (SNA) and (I) α2,3-sialylated glycans (MAIL-II) at steady state in epithelial cells (CD45^-^ cells) from *Mgat5*^-/-^ mice and *Mgat5*^WT^ controls. Median fluorescence intensity (MFI) was determined by flow cytometry. MFI were normalized for the average of *Mgat5*^-/-^ mice MFI. In the representative histograms, dark grey, light grey and orange depicts unstained control, *Mgat5^WT^* and *Mgat5^-/-^*, respectively. (J and K) Alcian Blue/periodic acid Schiff staining of the colonic tissue for goblet cells and mucus layer thickness analysis. (L) The mRNA expression levels at steady state of gene encoding for occludin in *Mgat5*^-/-^ and *Mgat5*^WT^ mice. Expression of target gene mRNAs was calculated based on housekeeping gene (*Gapdh*). mRNA expression levels were normalized for the average of mRNA levels of *Mgat5*^-/-^ mice. (D and E) n= 3 per group. (F) n = 14-19 per group. (G-I) n = 16–19 per group. (J) n= 3 per group. (K) n= 2 per group. (L) n= 14-19 per group. Each datapoint represents an individual animal. Data are represented as mean ± SD. ***p ≤ 0.001; ****p < 0.0001, using an unpaired two-tailed Student’s *t*-test.

**Supplementary Fig. 2. Deficiency in mucosa branched *N*-glycans leads to an impaired ILC3-IL-22 axis and gut inflammation.** (A) Gating strategy for different immune populations of colon lamina propria cells. Living cells were gated based on FVD staining and doublets were excluded using an FSC-H/FSC-A gate. ILC3s: CD45^+^, lineage negative, CD3^-^, CD90^+^; RORγt^+^; ILC1: CD45^+^, lineage negative, CD3^-^, CD90^+^, RORγt^-^, Tbet^+^, CD127^+^; CCR6^+^ILC3 (LTi-like): CD45^+^, lineage negative, CD3^-^, CD90^+^, EOMES^-^, CCR6^+^, Nkp46^-^, RORγt^+^; CCR6^-^NCR^-^RORyt^+^ILC3: CD45^+^, lineage negative, CD3^-^, CD90^+^, EOMES^-^, CCR6^-^, Nkp46^-^, RORγt^+^; Ex-ILC3/ILC1-like: CD45^+^, lineage negative, CD3^-^, CD90^+^, EOMES^-^, CCR6^-^, Nkp46^+^, RORγt^-^, Tbet^+^; NCR^+^RORyt^+^ILC3: CD45^+^, lineage negative, CD3^-^, CD90^+^, EOMES^-^, CCR6^-^, Nkp46^+^, RORγt^+^); T cells: CD45^+^, lineage negative, CD3^+^; Th17 cells: CD45^+^, lineage negative, CD3^+^, RORγt^+^; Treg: CD45^+^, lineage negative, CD3^+^, FOXP3^+^. For lineage negative, the following markers were used: B220, CD11c, Gr1, Ter119 and CD11b. Dendritic cells (DCs): CD45^+^, CD3^-^, CD11c^+^, CD11b^+^, MHC-II^+^, CD24^+^; Macrophages: CD45^+^, CD3^-^, CD11c^+^, CD11b^+^, MHC-II^+^, CD24^-^, CD64^+^; B cells: CD45^+^, CD3^-^, CD11c^-^, CD11b^-^, MHC-II^+^, CD24^+^; (B-H) Frequency of (B) T cells (CD3^+^) cells, (C) Th17 cells, (D) regulatory T cells, (E) B cells, (F) macrophages, (G) NCR^+^RORγt^+^ ILC3, and (H) dendritic cells in CD45^+^ cell population in *Mgat5*^WT^ and *Mgat5*^-/-^ mice at steady state. (I) Frequency of IL-22- producing cells in Th17 cells and (J) Mean fluorescence intensity (MFI) of intracellular IL22 in Th17 cells in *Mgat5*^WT^ and *Mgat5*^-/-^ mice at steady state. MFI were normalized for the average of *Mgat5*^-/-^ mice MFI. (K) Frequency of IL22-producing cells in NCR^+^RoRγt^+^ ILC3 and (L) Mean fluorescence intensity (MFI) of intracellular IL22 in NCR^+^RoRγt^+^ ILC3 in *Mgat5*^-/-^ and *Mgat5*^WT^ mice at steady state. MFI were normalized for the average of *Mgat5*^-/-^ mice MFI. (M) Frequency of IFNγ-producing cells in CD127^+^ILC1 cells and (N) Mean fluorescence intensity (MFI) of intracellular IFNγ in CD127^+^ILC1 cells in *Mgat5*^WT^ and *Mgat5*^-/^ mice at steady state. MFI were normalized for the average of *Mgat5*^-/-^ mice MFI. (O) Frequency of IFNγ-producing cells in Ex-ILC3/ILC1-like cells and (P) Mean fluorescence intensity (MFI) of intracellular IFNγ in Ex-ILC3/ILC1-like cells *Mgat5*^WT^ and *Mgat5*^-/-^ mice at steady state. MFI were normalized for the average of *Mgat5*^-/-^ mice MFI. The frequencies of different immune populations were determined by flow cytometry. (B) n = 8-11 per group. (C) n = 7–10 per group. (D) n = 6–7 per group. (E, F and H) n = 5 per group. (I, J) n=6-10 per group. (G, K, L, M, N, O and P) n=6-9 per group. Each datapoint represents an individual animal. Data is represented as mean ± SD. *p < 0.05 using an unpaired two-tailed Student’s *t*-test.

**Supplementary Fig. 3. Deficiency in mucosa branched *N*-glycans leads to an impaired ILC3-IL22 module after disease induction.**  Frequency of (A) ILC3, (B) CCR6^+^ILC3 (LTi-like cells) (C) Th17 cells, (D) CD3^+^ cells, (E) dendritic cells, (F) macrophages, and (G) B cells in CD45^+^ population in *Mgat5*^WT^ and *Mgat5*^-/-^ mice upon DSS-induced colitis. (H) Frequency of IL-22-producing cells in ILC3 and (I) Mean fluorescence intensity (MFI) of intracellular IL-22 in ILC3 in *Mgat5*^WT^ and *Mgat5*^-/-^ mice upon DSS-induced colitis. MFI were normalized for the average of Mgat5^-/-^ mice MFI. (J) Frequency of IL-22- producing cells in Th17 cells and (K) Mean fluorescence intensity (MFI) of intracellular IL22 in Th17 cells in *Mgat5*^WT^ and *Mgat5*^-/-^ mice upon DSS-induced colitis. MFI were normalized for the average of Mgat5^-/-^ mice MFI. (L) Frequency of IL-22-producing cells in NCR^+^RoRγt^+^ ILC3 and (M) Mean fluorescence intensity (MFI) of intracellular IL-22 in NCR^+^RoRγt^+^ ILC3 in *Mgat5*^WT^ and *Mgat5*^-/-^ mice upon DSS-induced colitis. MFI were normalized for the average of Mgat5^-/-^ mice MFI. (N-O) Frequency of (N) CD127^+^ILC1 and (O) Ex-ILC3/ILC1-like cells in CD45^+^ population in *Mgat5*^WT^ and *Mgat5*^-/-^ mice upon DSS-induced colitis. (P-S) Cytokine levels upon DSS-induced colitis in culture supernatants of colonic explants. Concentrations are normalized to tissue weight. The frequencies and MFIs of different immune populations were determined by flow cytometry. (A) n = 10-14 per group. (B) n = 4-5 per group (C, D) n = 9–13 per group. (E-G) n = 4–7 per group. (H-I) n = 14-15 per group. (J, K) n=11-10 per group. (L, M) n=8-10 per group. (N, O) n=6-9 per group. (P-S) n=6-9 per group. Each datapoint represents an individual animal. *p < 0.05; **p < 0.01; using an unpaired two-tailed Student’s t test or Mann-Whitney test.

**Supplementary Fig. 4. GlcNAc supplementation modulates immune response of *Mgat5^-/-^* mice rescuing ILC3-IL22 module**. (A) Frequency of CCR6^-^NCR^-^RoRγt^+^ ILC3, (B) NCR^+^RoRγt^+^ ILC3, and (C) Treg cells in CD45^+^ population in *Mgat5*^WT^, non-supplemented *Mgat5*^-/-^ mice and GlcNAc-supplemented. (D-F) Frequency of (D) Th17 cells, frequency of (E) IL-22- producing cells in Th17 cells and (F) Mean fluorescence intensity (MFI) of intracellular IL22 in Th17 cells in *Mgat5*^WT^, non-supplemented *Mgat5*^-/-^ mice and GlcNAc-supplemented. (G) Frequency of ILC3 in CD45*^+^* population in GlcNAc-supplemented and non-supplemented *Mgat5*^WT^ mice. (H) Frequency of IL-22-producing cells in ILC3 and (I) Mean fluorescence intensity (MFI) of intracellular IL22 in ICL3 in supplemented and non-supplemented *Mgat5*^WT^ mice with GlcNAc. (J-L) Frequency of (J) Th17, (K) Frequency of IL-22- producing cells in Th17 cells and (L) Mean fluorescence intensity (MFI) of intracellular IL22 in Th17 cells in supplemented and non-supplemented *Mgat5*^WT^ mice with GlcNAc. (M-P) Cytokine levels at steady-state in culture supernatants of colonic explants from supplemented and non-supplemented *Mgat5*^WT^ mice with GlcNAc. Concentrations are normalized to tissue weight. (Q-S) The mRNA expression levels at steady state of genes encoding for (Q) Il22bp, (R) Nod2 and (S) Fut2 in the colonic tissue measured by qPCR. Expression of target gene mRNAs was calculated based on housekeeping gene (*Gapdh*). mRNA expression levels were normalized for the average of mRNA levels of *Mgat5*^WT^ mice. The frequencies and MFIs of different immune populations were determined by flow cytometry. (A) n = 9-11 per group. (B and C) n = 9–11 per group. (D-E) n = 9–11 per group. (F, G and J) n = 7-8 per group. (H and I) n = 4 per group. (K-L) n = 4-5 per group. (M-P) n=4-10 per group. (Q-S) n= 7-7 per group. Each datapoint represents an individual animal. *p < 0.05; using an unpaired two-tailed Student’s t test or Mann-Whitney test.

**Supplementary Fig. 5. Glycan supplementation displays a protective effect against colitis development and restores the levels of SFCAs**. (A) Intestinal permeability measured by FITC-labelled dextran in *Mgat5*^WT^, non-supplemented *Mgat5*^-/-^ mice and GlcNAc-supplemented *Mgat5*^-/-^ mice. (B-E) The mRNA expression levels of genes encoding for (B) of claudin-2, (C) Gpr43, (D) Gpr109a and (E) Mct1 in the colonic tissue from in *Mgat5*^WT^, non-supplemented *Mgat5*^-/-^ mice and GlcNAc-supplemented *Mgat5*^-/-^ mice, measured by RT-qPCR. Expression of target gene mRNAs was calculated based on housekeeping gene (Gapdh). mRNA expression levels were normalized for the average of mRNA levels of Mgat5^-/-^ mice. (F) Principal component analysis (PCoA) of gut microbiota composition generated on Jaccard based on 16S rRNA sequencing of faecal samples from *Mgat5^WT^*, *Mgat5^-/-^* and GlcNAc-*s*upplemented *Mgat5^-/-^* mice at steady state. (A) n = 7 per group. (B-E) n = 9–16 per group. (F) n= 5 per group. Each datapoint represents an individual animal. Data is represented as mean ± SD. *p < 0.05; **p < 0.01; ***p < 0.001; using an unpaired two-tailed Student’s t test or Mann-Whitney test.

**Supplementary Fig. 6. Reduced *N*-glycan-related glycogene expression in CD patients is accompanied by reduced frequency of intestinal mucosal ILC3.** (A) The gene expression of a set of GlcNAc transferases *(MGAT5, MGAT5B, MGAT4D, MGAT4C, MGAT4B, MGAT4A, MGAT3, MGAT1)* in epithelial cells from inflamed and non-inflamed colonic tissue from CD patients. (B) Frequency of ILC3 in the inflamed and non-inflamed colonic tissue from CD patients (A, B) n=10-11 per group. Data were derived from Gene Expression Omnibus datasets (GSE134809). Wilcoxon test.
